# Flavin-Based Electron Bifurcation, Ferredoxin, Flavodoxin, and Anaerobic Respiration With Protons (Ech) or NAD^+^ (Rnf) as Electron Acceptors: A Historical Review

**DOI:** 10.3389/fmicb.2018.00401

**Published:** 2018-03-14

**Authors:** Wolfgang Buckel, Rudolf K. Thauer

**Affiliations:** ^1^Laboratory for Microbiology, Faculty of Biology, Philipps-Universität Marburg, Marburg, Germany; ^2^Max Planck Institute for Terrestrial Microbiology, Marburg, Germany

**Keywords:** electron bifurcation, ferredoxin, flavodoxin, electron-transferring flavoproteins (EtfAB), Rnf-complex, Ech-complex, energy conservation, crossed-over reduction potentials

## Abstract

Flavin-based electron bifurcation is a newly discovered mechanism, by which a hydride electron pair from NAD(P)H, coenzyme F_420_H_2_, H_2_, or formate is split by flavoproteins into one-electron with a more negative reduction potential and one with a more positive reduction potential than that of the electron pair. Via this mechanism microorganisms generate low- potential electrons for the reduction of ferredoxins (Fd) and flavodoxins (Fld). The first example was described in 2008 when it was found that the butyryl-CoA dehydrogenase-electron-transferring flavoprotein complex (Bcd-EtfAB) of *Clostridium kluyveri* couples the endergonic reduction of ferredoxin (E_0_′ = −420 mV) with NADH (−320 mV) to the exergonic reduction of crotonyl-CoA to butyryl-CoA (−10 mV) with NADH. The discovery was followed by the finding of an electron-bifurcating Fd- and NAD-dependent [FeFe]-hydrogenase (HydABC) in *Thermotoga maritima* (2009), Fd-dependent transhydrogenase (NfnAB) in various bacteria and archaea (2010), Fd- and H_2_-dependent heterodisulfide reductase (MvhADG-HdrABC) in methanogenic archaea (2011), Fd- and NADH-dependent caffeyl-CoA reductase (CarCDE) in *Acetobacterium woodii* (2013), Fd- and NAD-dependent formate dehydrogenase (HylABC-FdhF2) in *Clostridium acidi-urici* (2013), Fd- and NADP-dependent [FeFe]-hydrogenase (HytA-E) in *Clostridium autoethanogrenum* (2013), Fd(?)- and NADH-dependent methylene-tetrahydrofolate reductase (MetFV-HdrABC-MvhD) in *Moorella thermoacetica* (2014), Fd- and NAD-dependent lactate dehydrogenase (LctBCD) in *A. woodii* (2015), Fd- and F_420_H_2_-dependent heterodisulfide reductase (HdrA2B2C2) in *Methanosarcina acetivorans* (2017), and Fd- and NADH-dependent ubiquinol reductase (FixABCX) in *Azotobacter vinelandii* (2017). The electron-bifurcating flavoprotein complexes known to date fall into four groups that have evolved independently, namely those containing EtfAB (CarED, LctCB, FixBA) with bound FAD, a NuoF homolog (HydB, HytB, or HylB) harboring FMN, NfnB with bound FAD, or HdrA harboring FAD. All these flavoproteins are cytoplasmic except for the membrane-associated protein FixABCX. The organisms—in which they have been found—are strictly anaerobic microorganisms except for the aerobe *A. vinelandii*. The electron-bifurcating complexes are involved in a variety of processes such as butyric acid fermentation, methanogenesis, acetogenesis, anaerobic lactate oxidation, dissimilatory sulfate reduction, anaerobic- dearomatization, nitrogen fixation, and CO_2_ fixation. They contribute to energy conservation via the energy-converting ferredoxin: NAD^+^ reductase complex Rnf or the energy-converting ferredoxin-dependent hydrogenase complex Ech. This Review describes how this mechanism was discovered.

## Introduction

Electron bifurcation is a biochemical term for the splitting of hydride electron pairs into one electron with a more positive reduction potential and one with a more negative reduction potential than that of the electron pair. Via electron-bifurcation the reducing power of one electron is increased at the cost of that of the other electron. When operating in reverse, the mechanism is referred to as electron confurcating.

The best known electron bifurcation is that in the cytochrome *bc*_1_-complex of the respiratory chain using ubiquinol (ubiquinone/ubiquinol; E_0_′ = +90 mV) as hydride donor, Rieske iron-sulfur protein (E_0_′ = +285 mV) as the high reduction potential electron acceptor (electron acceptor 1) and cytochrome *b* (E_0_′ = −90 mV) as the low reduction potential electron acceptor (electron acceptor 2) (Brandt, 1996; Bergdoll et al., 2016). Ubiquinol-based electron bifurcation was first proposed by Peter Mitchell in 1975 (Mitchell, 1975a,b, 1976).

Only known since 2008, flavin-based electron bifurcation operates at reduction potentials about 400 mV [average of the two individual transitions (E1–E2)/2] more negative than ubiquinol-based electron bifurcation (Herrmann et al., 2008; Li et al., 2008). Enzymes that catalyze flavin-based electron bifurcating reactions typically are cytoplasmic and use NAD(P)H, F_420_H_2_, H_2_ or formate as hydride donor. Electron acceptor 1 can be NAD(P)^+^ (E_0_′ = −320 mV), methylene-tetrahydrofolate (−200 mV), pyruvate (−190 mV), the heterodisulfide CoM-S-S-CoB (−140 mV), caffeyl-CoA (−30 mV), crotonyl-CoA (−10 mV) or ubiquinone (+90 mV). Electron acceptor 2 is to date always a ferredoxin (Fd) or flavodoxin (Fld) (Buckel and Thauer, 2013) (Table 1).

**Table 1:** Electron-bifurcating flavoprotein complexes that have been characterized.

**Electron-bifurcating enzyme complexes**	**Hydride donor**	**Electron Acceptor 1**	**Electron acceptor 2**	**Cofactors**
Butyryl-CoA dehydrogenase-electron-transferring flavoprotein (Bcd-EtfAB)	2 NADH	Crotonyl-CoA	Fd/Fld	3 FAD
Caffeyl-CoA reductase-EtfAB (CarC-CarED)	2 NADH	Caffeyl-CoA	Fd	3 FAD; 2 [4Fe-4S]
Lactate dehydrogenase-EtfAB (LctD-LctCB)	2 NADH	Pyruvate	Fd	3 FAD; 1 [4Fe-4S]
UQ reductase-EtfAB (FixCX-FixBA)	2 NADH	Ubiquinone	Fld^*^	3 FAD; 2 [4Fe-4S]; riboflavin ?
Transhydrogenase (NfnAB)	2 NADPH	NAD^+^	Fd	1 FAD in NfnA; 1 FAD in NfnB; 1 [2Fe-2S]; 2 [4Fe-4S]
[FeFe]-Hydrogenase (HydABC)	2 H_2_	NAD^+^	Fd	1 FMN in HydB; 3 [2Fe2S]; 6 [4Fe-4S]; [**H**]-cluster
[FeFe]-Hydrogenase-CO_2_ reductase (HytABCDE-FdhA)	2 H_2_	NADP^+^	Fd	1 FMN in HytB; 3 [2Fe-2S]; 16 [4Fe-4S]; [**H**]-cluster; WSec
Formate dehydrogenase (HylABC-FdhF2)	2 HCOO^−^	NAD^+^	Fd	1 FMN in HylB; 4 [2Fe-2S]; 11 [4Fe-4S]; MoSec
[NiFe]-hydrogenase-heterodisulfide reductase (MvhADG-HdrABC)	2 H_2_	CoM-S-S-CoB	Fd	1 FAD in HdrA; 1 [2Fe2S]; 11 [4Fe-4S]; [NiFe]; 2 nc-[4Fe-4S]
Formate dehydrogenase-hetero-disulfide reductase (FdhAB-HdrABC)	2 HCOO^−^	CoM-S-S-CoB	Fd	1 FAD in HdrA; 1 FAD in FdhB; 1 [2Fe-2S]; 12 [4Fe-4S];Mo/WSec
F_420_H_2_-dependent heterodisulfide reductase (HdrA2B2C2)	2 F_420_ H_2_	CoM-S-S-CoB	Fd	1 FAD in HdrA; 1 [2Fe-2S]; 7 [4Fe-4S]
Methylene-H_4_ F reductase (MetFV-HdrABC-MvhD)	2 NADH	Methylene-H_4_F	?	2 FAD in HdrA; 1 [2Fe-2S]; 9 [4Fe-4S];2 FMN in MetFV

[H], H-cluster of [FeFe]-hydrogenases (Peters et al., 2015); Fd, ferredoxin from Clostridium pasteurianum; Fd/Fld, ferredoxin/flavodoxin from Acidaminococcus fermentans; Fld^*^, flavodoxin from Azotobacter vinelandii; nc, non-cubane; Yellow shading, Bcd, CarC, LctD, FixC, EtfA, and EtfB each contain a bound FAD. EtfB/FixA harbor the electron-bifurcating FAD. Blue shading, NfnB harbors the electron-bifurcating FAD; Green shading, complexes with HydB, HytB, or HylB are related to NuoF; Red shading, complexes with HdrABC.

Ferredoxins are low molecular mass (6–12 kDa), acidic iron-sulfur proteins with either one [2Fe-2S]-cluster or one, two, or more [4Fe-4S]-clusters (Johnson et al., 1982; Fukuyama, 2004). Ferredoxins characteristically transfer only one electron at a time with a reduction potential E_0_′ near −420 mV (Fitzgerald et al., 1980; Bianco et al., 1984; Smith et al., 1991), which is close to that of the hydrogen electrode at pH 7 (E_0_′ = −414 mV) (Thauer et al., 1977) and which is for stringency used in all our calculations (reactions 1–25). It should be noted, however, that the reduction potential of ferredoxins from some anaerobic microorganisms can be as low as −500 mV (e.g., Bengelsdorf et al., 2013; Li and Elliott, 2016) and as high as −340 mV (E_0_′2 of ferredoxin from *Acidaminococcus fermentans*) (Thamer et al., 2003). Although ferredoxins from different organisms differ significantly in primary structure and molecular mass, they can generally substitute for each other *in vitro*. For example, it was shown already in 1962, shortly after the discovery of ferredoxins, that [2Fe-2S]-ferredoxin from plant chloroplasts (12 kDa) functions *in vitro* as electron acceptor in the pyruvate: ferredoxin oxidoreductase reaction from *Clostridium pasteurianum*, and the 2x[4Fe-4S]-ferredoxin from *C. pasteurianum* (6 kDa) functions as electron donor in the ferredoxin: NADP^+^ reductase reaction in plant chloroplasts (Mortenson et al., 1962; Tagawa and Arnon, 1962).

In anaerobes growing with sufficient iron, mainly ferredoxins are found in general. However, when their cell growth is iron limited, flavodoxins (Fld) are instead preferentially synthesized (Knight and Hardy, 1966; Thamer et al., 2003). Flavodoxins are acidic FMN-containing one-electron-transferring proteins (14–23 kDa) with non-crossed-over reduction potentials: E_0_′ of the quinone (Q)/semiquinone couple (SQ) is more positive than that of the SQ/quinol (HQ) couple. E_0_′ of the SQ/HQ couple is generally more negative than −400 mV and that of the Q/SQ couple is at least 200 mV more positive (Alagaratnam et al., 2005). For example, the flavodoxin from *A. fermentans* exhibits reduction potentials of E_0_′ = −60 mV for the Q/SQ couple and E_0_′ = −420 mV for the SQ/HQ couple (Hans et al., 2002). Adding electrons to Q by decreasing the potential results in the formation of a stable neutral blue SQ until the potential falls beyond −240 mV when the HQ begins to build up.

Although flavodoxins and ferredoxins are structurally unrelated, they generally can substitute for each other. This does not mean that there is no selectivity at all for a specific ferredoxin or flavodoxin. Indeed, there is always some preference (Fitzgerald et al., 1980; Wan and Jarrett, 2002; Saen-Oon et al., 2015). For example, the catalytic efficiencies (k_cat_/K_m_) of the ferredoxin: NADP^+^ reductase from chloroplasts is much higher with the [2Fe-2S]-ferredoxin from the same plant than with the ferredoxin of other organisms or with flavodoxin (Paladini et al., 2009), and only with this ferredoxin the reductase can form a specific complex that can be crystallized (Kurisu et al., 2001). Many bacterial genomes carry and express several genes for different ferredoxins and flavodoxins and it has been shown that the different ferredoxins/flavodoxins are preferentially used *in vivo* by different enzymes (Peden et al., 2013; Li and Elliott, 2016).

The specificity of enzymes for a flavodoxin or ferredoxin, even if low, should be thought of when comparing literature data because usually only ferredoxin from *C. pasteurianum* was employed in the assays. The reason for this is that ferredoxin from *C. pasteurianum* is relatively easy to isolate and was until recently commercially available. It is therefore very likely that the catalytic efficiencies (*k*_cat_/*K*_m_) of electron-bifurcating enzymes would be considerably higher if tested with their physiological low reduction potential electron acceptor. And even more important, it is possible that there are some enzymes out there that are absolutely specific for a ferredoxin or a flavodoxin and would have no or only very low activity if assayed with ferredoxin from *C. pasteurianum*. This aspect should be considered in the search for new electron-bifurcating enzymes in spite of the fact that in the case of the electron-bifurcating butyryl-CoA dehydrogenase-EtfAB complex from *A. fermentans* there was no significant difference in the catalytic efficiency whether ferredoxin from *C. pasteurianum, C. tetanomorphum*, or flavodoxin from *A. fermentans* was used in the assays (Chowdhury et al., 2016).

This review will mainly discuss the work that led to the discovery of flavin-based electron bifurcation and how the pre-discovery of energy conservation via electron transport from ferredoxin to protons (Ech complex) and to NAD^+^ (Rnf complex) paved the way. For details on the individual electron-bifurcating enzymes complexes that have been purified and characterized (Table 1) the reader is referred to recent reviews on flavin-based electron bifurcation (Buckel and Thauer, 2013; Metcalf, 2016; Peters et al., 2016). Structural and mechanistic aspects are highlighted in Buckel and Thauer (in press). A short history of the discovery can be found in the prefatory chapter of *Annual Reviews of Microbiology 2015* (Thauer, 2015).

## Early findings: ferredoxin reduction with NAD(P)H

In 1969, in the laboratory of Karl Decker at the University of Freiburg in Germany, it was discovered that cell extracts of *Clostridium kluyveri* catalyzed the reduction of ferredoxin (Fd_ox_) with NADH (reaction 1) in an acetyl-CoA-dependent reaction (Thauer et al., 1969) and the reduction of Fd_ox_ with NADPH (reaction 2) in an NAD^+^-dependent reaction (Jungermann et al., 1969). In the assays NADH- and NADPH-regenerating systems were employed because the cell extracts also catalyzed both the reduction of acetyl-CoA with NADH to ethanol and butyrate, and the reduction of NAD^+^ with NADPH at relatively high specific rates.

(1) NADH + 2 Fd_ox_ → NAD^+^ + 2 ${\text{Fd}}_{\text{red}}^{-}$ + H^+^ (acetyl-CoA dependent; catalyzed by cell extracts) Δ*G*°′ near +20 kJ/mol NAD(P)H
(2) NADPH + 2 Fd_ox_ → NADP^+^ + 2 ${\text{Fd}}_{\text{red}}^{-}$ + H^+^ (NAD^+^ dependent; catalyzed by cell extracts) Δ*G*°′ near +20 kJ/mol NAD(P)H

The enzymes catalyzing reactions 1 and 2 had been searched for, because *C. kluyveri* stoichiometrically forms H_2_ when growing on ethanol and acetate (reaction 3, simplified version) (Barker et al., 1945; Thauer et al., 1968b).

(3) 6 Ethanol + 4 Acetate^−^ → 5 Butyrate^−^ + H^+^ + 2 H_2_ + 4 H_2_O
  Δ*G*°′ = −180 kJ/mol H^+^

In this fermentation ethanol had been shown to be oxidized to acetyl-CoA via NAD-specific ethanol dehydrogenase and NAD(P)-dependent acetaldehyde dehydrogenase (Burton and Stadtman, 1953). Thus only NAD(P)H appeared available as electron donors for H_2_ formation, and it was known that in clostridia H_2_ is generated by proton reduction with reduced ferredoxin (reaction 4), which is catalyzed by a cytoplasmic hydrogenase (Fredricks and Stadtman, 1965a,b)

(4) 2 ${\text{Fd}}_{\text{red}}^{-}$ + 2 H^+^ ⇋ 2 Fd_ox_ + H_2_
  Δ*G*°′ near 0 kJ/mol

Under standard conditions the reduction of ferredoxin (E_0_′ near −420 mV) with NAD(P)H (E_0_′ = −320 mV) is an endergonic reaction (reactions 1 and 2). At first glance, this was not a problem since it is accepted that reduction potential differences of 100 mV under standard conditions can be overcome *in vivo* under physiological conditions, where the substrate- and product concentrations are not 1 M or 10^5^ Pa (gases) as under standard conditions. For example, the reduction of NAD^+^ with malate (E_0_′ = −170 mV) in the Krebs cycle is textbook knowledge. In cell extracts the thermodynamic problem of ferredoxin reduction with NAD(P)H was easy to overcome by starting the reaction in the absence of H_2_ and by keeping the NAD(P)H/NAD(P)^+^ ratio high via an exergonic regenerating system. *In vivo*, however, the situation is different. H_2_-gas with only a few percent CO_2_, continuously bubbles out of growing *C. kluyveri* cultures, which indicates that the reduction potential of the 2 H^+^/H_2_ couple is near −400 mV. To reach this reduction potential, the NAD(P)H/NAD(P)^+^ ratio within the cell would have to be almost 1,000:1. Such a high ratio could be obtained in the CoA-acetylating acetaldehyde dehydrogenase reaction with a standard reduction potential (E_0_′) of the acetyl-CoA/acetaldehyde couple near −400 mV but not in the ethanol dehydrogenase reaction with E_0_′ of the acetaldehyde/ethanol couple of −200 mV. Measurements of the intracellular NAD(P)H and NAD(P)^+^ concentrations showed that the NADH to NAD^+^ ratio was near 1:4 and the NADPH/NADP^+^ ratio was near 1.5:1 (Decker and Pfitzer, 1972).

To address these problems some alternative hypotheses were made. One hypothesis was that the enzyme catalyzing the reduction of ferredoxin with NAD(P)H in *C. kluyveri in vivo* forms a tight complex with the NAD(P)H-regenerating acetaldehyde dehydrogenase and thus forms a micro-compartment, in which the NAD(P)H/NAD(P)^+^ ratio would be as high as 1,000:1. The hypothesis was based on the finding that ethanol- and acetaldehyde dehydrogenase in *C. kluyveri* are located in a micro-compartment (Hillmer and Gottschalk, 1972, 1974; Lurz et al., 1979; Seedorf et al., 2008). Alternatively, it was proposed that *C. kluyveri* contains a second acetaldehyde dehydrogenase that directly reduces ferredoxin (Schoberth and Gottschalk, 1969). An enzyme that catalyzes the oxidation of acetaldehyde to acetate (E_0_′ = −580 mV) had previously been discovered *in* the S-organism of *Methanobacillus omelianskii* (Brill and Wolfe, 1966; Reddy et al., 1972).

The acetyl-CoA dependence of the NADH: ferredoxin reductase activity in cell extracts was interpreted as indicating that ferredoxin reduction with NADH is allosterically regulated by the acetyl-CoA/CoA couple (Jungermann et al., 1971b), which made sense since acetyl-CoA and NADH are at metabolic branch points in the energy metabolism of *C. kluyveri* (reaction 3). For every acetyl-CoA generated from ethanol that is converted via acetyl-phosphate to acetate and not via reduction with NADH to butyrate, two H_2_ have to be formed from NADH. The role model was pyruvate carboxylation in the liver of animals. This reaction is allosterically regulated by acetyl-CoA to ascertain that pyruvate oxidation to acetyl-CoA and pyruvate carboxylation to oxaloacetate are balanced for the citric acid cycle to function (Scrutton et al., 1965). Acetyl-CoA-dependent NADH: ferredoxin oxidoreductase activity was also found in other butyrate- forming anaerobes (Jungermann et al., 1971a, 1973, 1974; Petitdemange et al., 1973) and allosteric regulation by acetyl-CoA was also an attractive interpretation for their fermentations (Thauer et al., 1977). Similarly, it was considered that the reduction of ferredoxin with NADPH is allosterically regulated by the redox charge (NAD^+^/NADH couple) (Thauer et al., 1971). The two papers on the allosteric regulation of ferredoxin reduction (Jungermann et al., 1971b; Thauer et al., 1971) were published in the *Journal of Biological Chemistry*, which at that time was the most prestigious journal in biochemical sciences. In the early 1970s allosteric regulation (Stadtman, 1966) was still a hot topic.

Indirect evidence for ferredoxin reduction with NADH also came from studies of glutamate fermentation by *A. fermentans* (reaction 5) by Wolfgang Buckel in the laboratory of Horace A. Barker at the University of California in Berkeley (Buckel and Barker, 1974). It was sown that the fermentation proceeds via 2-hydroxyglutaryl-CoA, glutaconyl-CoA and crotonyl-CoA followed by the disproportionation of 5 crotonyl-CoA to 6 acetyl-CoA and 2 butyryl-CoA (see Figure 14B in Buckel and Thauer, 2013). It appeared to only involve NAD-dependent redox reactions and it was proposed that H_2_ formation was somehow coupled to crotonyl-CoA disproportionation, a reaction also catalyzed by *C. kluyveri* (Thauer et al., 1968a); see Figure 2 in Buckel and Barker (1974). However, Wolfgang Buckel first focused on the two reactions with unusual mechanisms, namely on the dehydration of 2-hydroxyglutaryl-CoA to glutaconyl-CoA (Schweiger and Buckel, 1984); for a review see Buckel et al. (2012), and on the decarboxylation of glutaconyl-CoA to crotonyl-CoA (Buckel and Semmler, 1982); for a review see Buckel (2001). His interest in ferredoxin reduction with NADH had to wait until somewhat later (Härtel and Buckel, 1996).

(5) 5 Glutamate^−^ + 6 H_2_O + 2 H^+^ → 5 ${\text{NH}}_{4}^{+}$ + 5 CO_2_ + 6 Acetate^−^ + 2 Butyrate^−^ + H_2_
  ΔG°′ = −317 kJ/mol H_2_

“Do not believe something because you can explain it” is the third of Arthur Kornberg's “ten biochemical commandments” (Kornberg, 2000). To determine whether the enzymes catalyzing reactions 1 and 2 in *C. kluyveri* are indeed allosterically regulated, their purification was necessary. However, several attempts in the early 1970s failed for reasons that will become clear further below. Therefore, the molecular and catalytic properties of the two “ferredoxin reductases” remained largely unknown until almost 40 years later, when they were finally purified. It was found that the dependence on acetyl-CoA and NAD^+^, respectively, was due to their electron-bifurcation mechanism and not to allosteric regulation. The “NADH: ferredoxin reductase” (reaction 1) was the first electron-bifurcating flavoenzyme to be characterized (Li et al., 2008) and the “NADPH: ferredoxin reductase” (reaction 2) was the third one (Wang et al., 2010). But before this could happen, it had to be realized that ferredoxin reduction with NAD(P)H *in vivo* is only possible when coupled to an exergonic reaction and that a coupling mechanisms for this had to be found.

## The thermodynamic conundrum

The oxidation of acetaldehyde either to acetyl-CoA or acetate with ferredoxin as electron acceptor was an attractive hypothesis to explain ferredoxin-dependent H_2_ formation in *C. kluyveri* when growing on ethanol and acetate. This explanation, however, did not hold for growth on crotonate which is fermented by *C. kluyveri* to acetate, butyrate and H_2_ bubbling out of the culture (reaction 6) (Thauer et al., 1968a).

**Figure 1:**
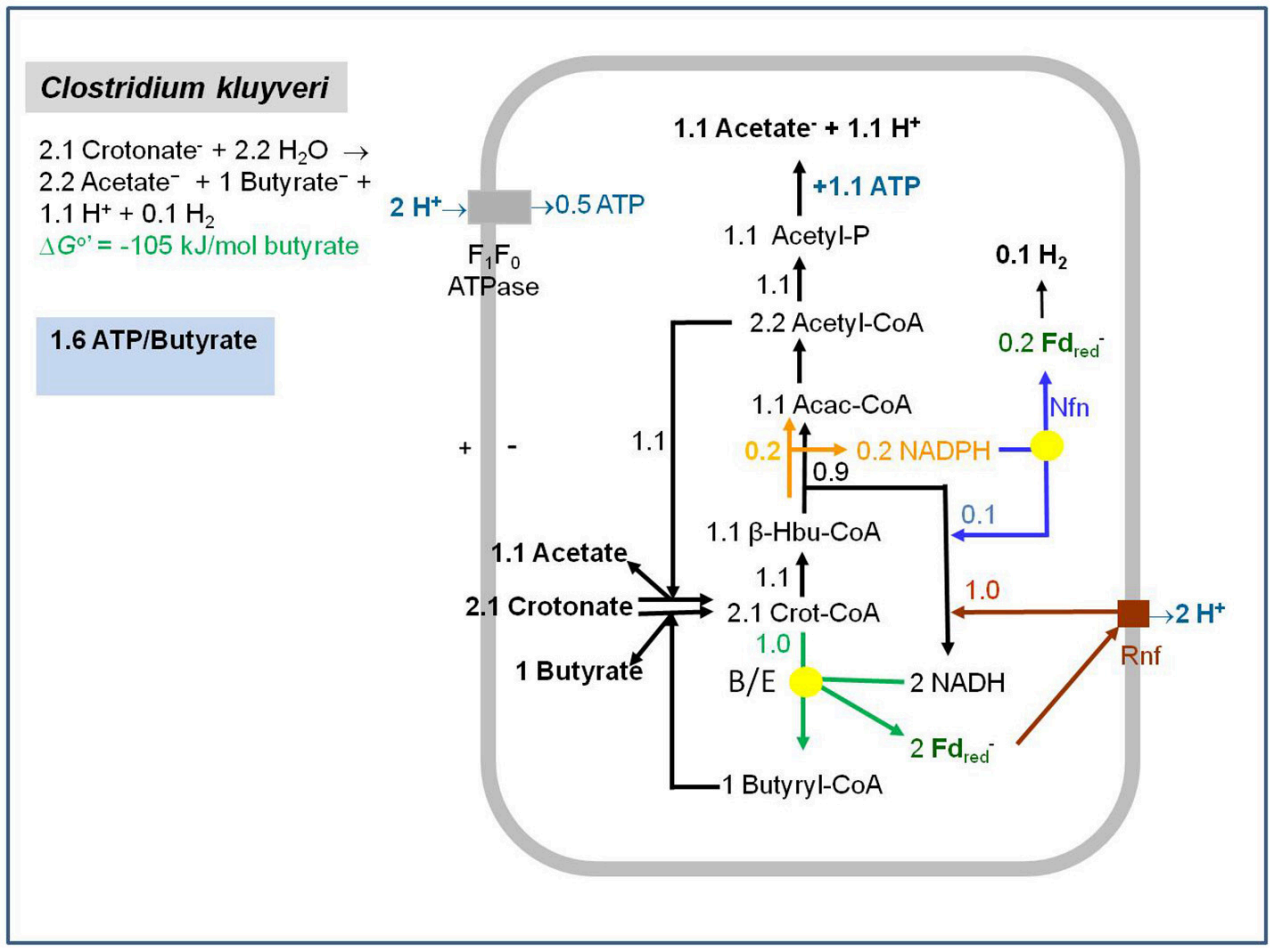
Energy metabolism of *Clostridium kluyveri* growing in batch culture on crotonate. For simplification, acetyl-CoA and reduced ferredoxin used in biosyntheses are not considered. The yellow dots represent the electron-bifurcating butyryl-CoA dehydrogenase-EtfAB (B/E) complex and the NfnAB (Nfn) complex. Acac-CoA, acetoacetyl-CoA; β-Hbu-CoA, beta-hydroxybutyryl-CoA; Crot-CoA, crotonyl-CoA; acetyl-P, acetyl-phosphate. *C. kluyveri* contains an NAD-specific and an NADP-specific (orange arrow) β-hydroxybutyryl-CoA dehydrogenase (Madan et al., 1973). The metabolic scheme is compatible with the finding that H_2_-formation by cell suspensions of *C. kluyveri* is inhibited by the protonophore tetrachlolorosalicylanilide (TCS) and the inhibition is relieved by dicyclohexylcarbodiimide (DCCD), an inhibitor of the proton-translocating membrane ATPase (Pfeiff, 1991). In the presence of TCS, the F_1_F_0_-ATPase hydrolyzes ATP to prevent the collapse of the electrochemical proton potential and as a consequence of ATP hydrolysis also the acetyl-CoA and acetyl-phosphates pools are depleted inhibiting acetyl-CoA reduction to butyryl-CoA. In the presence of DCCD, ATP hydrolysis via the F_1_F_0_ ATPase is stopped.

(6) 2.1 Crotonate^−^ + 2.2 H_2_O → 2.2 Acetate^−^ + Butyrate^−^ + 1.1 H^+^ + 0.1 H_2_
  ΔG°′ = −105 kJ/mol butyrate (see Figure 1)

The fermentation involves the oxidation of 3-hydroxybutyryl-CoA to acetoacetyl-CoA (E_0_′ = −250 mV) with NAD(P)^+^ and the reduction of crotonyl-CoA to butyryl-CoA (E_0_′ = −10 mV) with NADH but not the oxidation of acetaldehyde. H_2_ formation from NAD(P)H would require a 3-hydroxybutyryl-CoA/acetoacetyl-CoA ratio in the cells of more than 10^5^:1. An even higher ratio is predicted by the finding that *C. kluyveri* assimilates acetate and CO_2_ into cell carbon during growth mainly via reduction of acetyl-CoA + CO_2_ to pyruvate (E_0_′ = −500 mV) with ferredoxin as electron donor (Andrew and Morris, 1965; Decker et al., 1966; Thauer et al., 1968a; Gottschalk and Chowdhury, 1969). A reduction potential of −500 mV would require a 3-hydroxybutyryl-CoA/acetoacetyl-CoA ratio of more than10^8^, which is thermodynamically and kinetically completely unrealistic. These reflections clearly indicated that ferredoxin reduction with NAD(P)H in *C. kluyveri* must somehow be coupled to an exergonic reaction.

One possibility was that ferredoxin reduction with NADH (reaction 1) is chemiosmotically coupled to the exergonic reduction of crotonyl-CoA to butyryl-CoA (E_0_′ = −10 mV) with NADH (reaction 7), which is an intermediate step in butyrate formation from ethanol and acetate (reaction 3) or from crotonate (reaction 6). As early as 1964 reaction 7 was shown to be catalyzed in cell extracts of *Megasphaera elsdenii* (formerly *Peptostreptococcus elsdenii*) by an electron-transferring flavoprotein and butyryl-CoA dehydrogenase (Baldwin and Milligan, 1964). In *Escherichia coli* the analogous reduction of fumarate to succinate (E_0_′ = +33 mV) with NADH is catalyzed by a membrane-associated, cytochrome *b*-containing enzyme complex, involves menaquinone (E_0_′ = −70 mV) and is coupled with electron-transport phosphorylation (Krebs, 1937; Hirsch et al., 1963) for a review see Iverson et al. (1999). Therefore, such a coupling mechanism was considered for the crotonyl-CoA reduction to butyryl-CoA, inspired by a preliminary report of ATP production coupled to crotonyl-CoA reduction with NADH (Gunsalus and Schuster, 1961). This idea was challenged, however, when it was found that butyryl-CoA dehydrogenase was present mainly in the cytoplasm. A chemiosmotic coupling of the endergonic reduction of ferredoxin with NADH to the exergonic reduction of crotonyl-CoA with NADH thus did not appear likely.

(7) NADH + H^+^ + crotonyl-CoA → NAD^+^ + butyryl-CoA (catalyzed by cell extracts but not by the purified butyryl-CoA dehydrogenase)
  Δ*G*°′ near −60 kJ/mol

Although the enzyme activities catalyzing reactions 1, 2, and 7 in *C. kluyveri* were found in the soluble cell fraction, this did not completely exclude an association with the cytoplasmic membrane, from which the proteins could have been sheared or fallen off when the cell extract were prepared. Indeed, the acyl-CoA dehydrogenases involved in β-oxidation of fatty acids in mitochondria and aerobic bacteria (Bennett and Rudolph, 1995; Kunau et al., 1995) are only loosely connected via the electron-transferring flavoproteins EtfAB to the EtfAB: quinone oxidoreductase in the membrane (Watmough and Frerman, 2010). Therefore, there was still the possibility that ferredoxin reduction with NADH is somehow driven by the electrochemical proton- or sodium ion potential that is built up by the reduction of crotonyl-CoA to butyryl-CoA or, in the case of *A. fermentans*, by the decarboxylation of glutaconyl-CoA to crotonyl-CoA catalyzed by an integral membrane protein, for which there was some evidence. Thus, the formation of H_2_ from ethanol and acetate in cell suspensions of *C. kluyveri* was not sodium ion dependent, was inhibited by the protonophore tetrachlolorosalicylanilide and the inhibition was relieved by dicyclohexylcarbodiimide, an inhibitor of the proton translocating membrane ATPase (Pfeiff, 1991) (see also Figure 1). And it was shown with vesicles of *A. fermentans* that glutaconate-dependent H_2_ formation was effectively inhibited by protonophores, sodium ionophores and valinomycin. As a control, H_2_ formation from pyruvate was not affected by these compounds (Härtel and Buckel, 1996).

## A paradigm shift: electron-transport phosphorylation with protons as electron acceptor

The 1977 review on “Energy conservation in chemotrophic anaerobic bacteria” by Thauer et al. (1977) (still cited almost 100 times per year) summarized evidence that many strict anaerobes can conserve energy via electron-transport phosphorylation using electron acceptors such as nitrate, nitrite, fumarate, sulfate, CO_2_, and possibly elemental sulfur and Fe(III). These electron acceptors had in common reduction potentials that were much more positive than that of the NAD(P)^+^/NAD(P)H couple (E_0_′ = −320 mV), and the electron- transport chains, as far as known, involved cytochromes and menaquinone (E_0_′ = −70 mV). Therefore, it was thought that anaerobes, such as Clostridia that did not contain cytochromes and menaquinone conserved energy only via substrate-level phosphorylation. Already during the writing of that review, this paradigm had changed.

In 1976 the first evidence was published that electron-transport phosphorylation is possible between redox components with reduction potentials more negative than those of the NAD(P)^+^/NAD(P)H couple. Robert Uffen at Michigan State University in East Lansing, USA, reported that a newly isolated *Rhodopseudomonas* species (later named *Rubrivax gelatinosus*) can ferment CO + H_2_O to CO_2_ and H_2_ (reaction 8) and couple this reaction with the synthesis of ATP (reaction 9), as evidenced by growth of the phototrophic bacterium on CO in the dark (Uffen, 1976). This “water-gas shift reaction” was catalyzed by the membrane fraction and involved neither cytochromes nor quinones (Wakim and Uffen, 1983).

(8) CO + H_2_O ⇋ CO_2_ + H_2_ + ΔμH^+^
(9) ADP + Pi + ΔμH^+^ ⇋ ATP + H_2_O

Ten years later the group of Paul Ludden at the University of Wisconsin in Madison, USA showed that in *Rhodospirillum rubrum* growing chemotrophically on CO (Kerby et al., 1995) the genes *cooFS* and *cooMKLXUH* are involved in the conversion of CO to H_2_ and CO_2_ (reaction 8). Kerby et al. (1992), Fox et al. (1996), and Singer et al. (2006): *cooS* encodes a nickel-containing CO-dehydrogenase, *cooF* a polyferredoxin and *cooH* a [NiFe]-hydrogenase. CooL, CooX, and CooU are iron-sulfur proteins and CooK and CooM are integral membrane proteins. The eight proteins form a tight membrane-associated complex with the active site-harboring subunits CooS and CooH facing the cytoplasm. Such a complex was also found in non-phototrophic bacteria, e.g., in *Carboxythermus hydrogenoformans* growing on CO (Soboh et al., 2002).

*Methanosarcina barkeri* also catalyzes reaction 8 as shown in the laboratory of Rudolf Thauer at the Philipps Universität Marburg, Germany (Bott et al., 1986; Bott and Thauer, 1989). The reaction is, however, mediated by a cytoplasmic CO dehydrogenase (reaction 10), ferredoxin, and a membrane-associated [NiFe]-hydrogenase complex (reactions 11). The hydrogenase complex is composed of six different subunits, EchA-E, which are homologous to CooMKLXUH in the CO-hydrogen lyase complex (Künkel et al., 1998; Meuer et al., 1999, 2002). Ech was chosen as acronym to indicate that the EchA-E subunits have sequence similarities to subunits of ***E***. ***c**oli* **h**ydrogenase-3. The subunits are also homologous to subunits of the energy-converting NADH dehydrogenase from *E. coli*, the reason why the hydrogenase is also referred to as **e**nergy-**c**onverting **h**ydrogenase. Indeed, the reduction of protons with reduced ferredoxin to H_2_ was found to be coupled with the build-up of an electrochemical proton potential (ΔμH^+^) or sodium ion potential (ΔμNa^+^) (reaction 11) (Hedderich and Forzi, 2005; Thauer et al., 2010).

(10) CO + H_2_O + 2 Fd_ox_ ⇋ CO_2_ + 2 H^+^ + 2 ${\text{Fd}}_{\text{red}}^{-}$
(11) 2 ${\text{Fd}}_{\text{red}}^{-}$ + 2 H^+^ ⇋ 2 F_dox_ + H_2_ + ΔμH^+^/ ΔμNa^+^

EchA-F from *M. barkeri* differs from the energy-converting hydrogenase CooHKLMUX from *R. rubrum* and *C. hydrogenoformans* in not forming a tight complex with its ferredoxin and CO dehydrogenase. A stable complex probably does not form because the reduced ferredoxin, generated by the energy-converting hydrogenase in methanogens is used in electron transfer to more than one oxidoreductase, e.g., also to formylmethanofuran dehydrogenase (Thauer et al., 2010).

Ech-type hydrogenases were later also found in many other anaerobes (Thauer et al., 2010), e.g., some clostridia (Calusinska et al., 2010; Biswas et al., 2015), sulfate reducing bacteria (Pohorelic et al., 2002; Rodrigues et al., 2003), some acetogenic bacteria (Wang et al., 2013b), methanogenic archaea (Tersteegen and Hedderich, 1999), *Pyrococcus furiosus* (Sapra et al., 2003), *Thermoanaerobacter tengcongensis* (Soboh et al., 2004) and syntrophic bacteria (Manzoor et al., 2016), some of which are devoid of CO dehydrogenase.

An energy-converting hydrogenase complex was not found in *C. kluyveri* (Seedorf et al., 2008) and *A. fermentans* (Chang et al., 2010). Instead, in these two model organisms an energy converting ferredoxin: NAD^+^ reductase complex was found; such a complex was first discovered in nitrogen fixing bacteria 17 years after anaerobic growth on carbon monoxide was discovered (Uffen, 1976).

## Electron-transport phosphorylation with NAD^+^ as electron acceptor

In 1993, in the laboratories of Katzuhiko Saeki at Osaka University, Japan, and of Werner Klipp at Ruhr University Bochum, Germany, six genes discovered in *Rhodobacter capsulatus* were predicted to encode for components of a membrane-associated electron-transport system to nitrogenase (Saeki et al., 1993; Schmehl et al., 1993). The deduced amino acid sequences of the genes *rnfABCGEH* (**R**hodobacter **n**itrogen **f**ixation) were later found out to show similarities to those of subunits of the respiratory sodium ion-translocating NADH-dehydrogenase complex from *Vibrio* sp. (Jouanneau et al., 1998; Backiel et al., 2008). Based on sequence comparisons and membrane topologies, the putative Rnf complex was expected to catalyze the sodium ion motive force-driven electron transport from NADH to ferredoxin (reaction 12), which is required for nitrogen fixation (Kumagai et al., 1997; Saeki and Kumagai, 1998; Jeong and Jouanneau, 2000; Desnoues et al., 2003). The *rnf* genes were shown to be involved also in nitrogen fixation in other diazotrophic bacteria including *Pseudomonas stutzeri* (Desnoues et al., 2003) and *Azotobacter vinelandii* (Ledbetter et al., 2017). The Rnf complexes from these nitrogen-fixing bacteria still have not been biochemically characterized.

(12) NADH + 2 Fd_ox_ + ΔμH^+^/Na^+^ ⇋ NAD^+^ + 2 ${\text{Fd}}_{\text{red}}^{-}$ + H^+^

In 2005, the group of Wolfgang Buckel at the Philipps University Marburg, Germany, provided the first evidence that a membrane-associated complex catalyzing reaction 12 operates in a bacterium that does not fix nitrogen, namely *Clostridium tetanomorphum* growing on glutamate (Boiangiu et al., 2005). Membrane preparations catalyzed the oxidation of NADH by ferricyanide. Using this reaction as spectrophotometric assay the enzyme was solubilized with dodecylmaltoside and purified by column chromatographies under anoxic conditions until SDS-PAGE revealed 6 discrete protein bands, the N-termini of which matched those derived from the putative *rnfABCDEG* genes of the sequenced genome of *Clostridium tetani* (Brüggemann et al., 2003). The cloned and sequenced six encoding genes showed, on the level of protein, sequence similarity to the *rnf* genes in *R. capsulatus*. Subsequent work revealed that the complex catalyzed a sodium ion-dependent reversible reduction of NAD^+^ with ferredoxin (reverse reaction 12) in *Acetobacterium woodii* (Biegel and Müller, 2010; Hess et al., 2013) and in *A. fermentans* (Chowdhury et al., 2016). It most probably translocates protons in *Clostridium ljungdahlii* (Tremblay et al., 2013) and *C. kluyveri* (Pfeiff, 1991; Seedorf et al., 2008). The stoichiometry is most likely one sodium ion or proton translocated per electron.

Genes for the Rnf complex have in the meantime been found in many other strict anaerobes, e.g. *Clostridium sticklandii* (Fonknechten et al., 2010), *Thermoanaerobacterium saccharolyticum* (Tian et al., 2016), acetogenic bacteria (Imkamp et al., 2007; Müller et al., 2008; Biegel and Müller, 2010; Köpke et al., 2010; Tremblay et al., 2013), butyribacteria (Hackmann and Firkins, 2015), *Acholeplasma* spp. (Kube et al., 2014), *Syntrophus aciditrophicus* (McInerney et al., 2007), sulfate reducers (Strittmatter et al., 2009; Pereira et al., 2011), acetoclastic methanogens (Schlegel et al., 2012) and methanotrophic archaea (Wang et al., 2014).

The presence of the Rnf complex in *C. kluyveri* did not solve the question of how ferredoxin is reduced with NADH in the fermentation of ethanol and acetate (reactions 3). Under physiological conditions, where the concentrations of non- gaseous substrates and products are more like 1 mM rather than 1 M, the free energy change Δ*G*′ associated with reaction 3 is only near −95 kJ/mol H^+^ rather than −180 kJ/mol H^+^. This allows the synthesis of about 1.5 ATP, considering that about −70 kJ/mol are required for ADP phosphorylation *in vivo* (Thauer et al., 1977), of which 1 ATP is formed by substrate level phosphorylation (Thauer et al., 1968b). The ATP gain of 1.5 is consistent with the growth yield of 10 g/mol H^+^ (Thauer et al., 1968b) taking into account that *C. kluyveri* grows on acetate and CO_2_ as sole carbon source with a theoretical Y_ATP_ of near 7 g/mol (Thauer et al., 1977). From these data, it was predicted that the Rnf complex in *C. kluyveri* catalyzes *in vivo* the reduction of NAD^+^ with ferredoxin, thereby conserving the free-energy change in an electrochemical proton potential (reaction 12) that is required to drive the phosphorylation of about 0.5 mol ATP. If the Rnf complex would be involved in ferredoxin reduction, this would be at the expense of ATP hydrolysis (back reaction 9), which is not supported by the growth yield data. Thus, how in *C. kluyveri* ferredoxin is reduced remained elusive.

Similar arguments hold true for the fermentation of glutamate by *A. fermentans* (reaction 5). At 1 mM concentrations of substrates and non-gaseous products, reaction 5 is associated with a free energy change of −420 kJ/mol H_2_ rather than −317 kJ/mol H_2_. This free energy change is sufficient to drive the phosphorylation of at least 6 ATP (see above), of which 3 are provided by substrate- level phosphorylation and 2.5 by the sodium-motive force generated in the decarboxylation reaction associated with the conversion of 5 glutaconyl-CoA to 5 crotonyl-CoA (see Figure 14 in Buckel and Thauer, 2013). Thus, from the thermo-dynamically predicted ATP gain of 6, only 5.5 could be mechanistically accounted for. The discovery of the Rnf complex in *A. fermentans* allowed the ATP gap to be closed, but only if it was assumed that the complex *in vivo* catalyzes the exergonic reduction of NAD^+^ with ferredoxin rather than the reverse reaction 12. Therefore, the question of how ferredoxin is reduced in *A. fermentans* also remained open.

## Solving the enigma: the EtfAB-butyryl-CoA dehydrogenase complex is electron bifurcating

In 2006, the groups of Wolfgang Buckel and Rudolf Thauer teamed up with the group of Gerhard Gottschalk at the University of Göttingen, Germany, to sequence the genome of *C. kluyveri* (Seedorf et al., 2008). They hoped to gain information on the topology of the proteins involved in H_2_ formation (reactions 1 and 2) and crotonyl-CoA reduction (reaction 7) from the genome sequence. Since 1964 biochemical evidence had accumulated that the reduction of crotonyl-CoA to butyryl-CoA with NADH in butyric acid forming bacteria involves a complex of butyryl-CoA dehydrogenase (Bcd) with the **e**lectron-**t**ransferring **f**lavoproteins EtfAB (Baldwin and Milligan, 1964; Herrmann et al., 2008). Gene clusters predicted to encode Bcd and EtfAB were found in the genome of *C. kluyveri* but not a gene for an EtfAB: quinone oxidoreductase, with which acyl-CoA dehydrogenases in complex with EtfAB are docked to the membrane in aerobic bacteria and mitochondria (Watmough and Frerman, 2010). The genome also did not harbor genes for menaquinone or ubiquinone biosynthesis (Seedorf et al., 2008). The cytoplasmic location of the butyryl-CoA-EtfAB complex was confirmed by immune gold labeling showing that butyryl-CoA dehydrogenase was present mainly (90%) in the cytoplasm (Herrmann, 2008). A chemiosmotic coupling of ferredoxin reduction with NAD(P)H to crotonyl-CoA reduction with NADH could therefore no longer be considered.

Comparison of the amino acid sequences of the Etf proteins of butyrate-forming anaerobes with those of Etf and Etf-like proteins of respiring bacteria (Boynton et al., 1996) indicated that, “while homology occurs with the mitochondrial ETF and bacterial ETFs, the greatest similarity is with the putative ETFs from clostridia and with *fixAB* gene products from nitrogen-fixing bacteria” (O'Neill et al., 1998). In 2004, Thomas Edgren and Stefan Nordlund at the Stockholm University, Sweden, finally proposed that “the *fixABCX* genes *in R. rubrum* encode a putative membrane complex participating in electron transfer to nitrogenase” (Edgren and Nordlund, 2004). An EtfAB phylogenetic tree shows that Etfs involved in fatty acid oxidation form one group (group I) whilst Etfs involved in crotonyl-CoA reduction and in nitrogen fixation form a second group (group IIa and group IId1, respectively) (Costas et al., 2017). Whereas group II Etfs exhibit NADH dehydrogenase activity and contain an FAD bound each in EtfA and EtfB, those of group I lack this activity and contain an AMP instead of an FAD bound in EtfB.

All these findings and the acetyl-CoA dependence of ferredoxin reduction with NADH in cell extracts of butyric acid forming bacteria (Thauer et al., 1969) (reaction 1) prompted Wolfgang Buckel in 2007 to propose that crotonyl-CoA reduction with NADH is ferredoxin dependent (reaction 13) (Herrmann et al., 2008). His group had previously noted that, whereas the cell extracts that contained ferredoxin and ferredoxin-dependent hydrogenase catalyzed the reduction of crotonyl-CoA (E_0_′ = −10 mV) with NADH (E_0_′ = −320 mV), the purified EtfAB-Bcd complex no longer catalyzed this reaction, although it did catalyze the oxidation of butyryl-CoA with ferricenium hexafluorophosphate as artificial electron acceptor, the assay with which purification of the complex was followed. It was then indeed shown that the purified EtfAB-Bcd complex from *C. kluyveri* catalyzes the crotonyl-CoA-dependent reduction of ferredoxin (E_0_′ = −420 mV) with NADH forming butyryl-CoA in the stoichiometry given in reaction 13. The specific activity of the purified complex was compatible with the specific rates of butyrate formation in growing cultures (Li et al., 2008). After almost 40 years the answer to the question of what drives ferredoxin reduction with NADH, unfolded before our eyes.

(13) 2 NADH + crotonyl-CoA + 2 Fd_ox_ → 2 NAD^+^ + butyryl-CoA + 2 ${\text{Fd}}_{\text{red}}^{-}$
  ΔG°′ near −40 kJ/mol butyryl-CoA

Reaction 13 is essentially irreversible *in vivo*. In the butyrate-oxidizing anaerobe *Syntrophomonas wolfei*, the oxidation of butyryl-CoA with NAD^+^ to crotonyl-CoA is catalyzed by a membrane- associated complex energized by the proton motive force (Müller et al., 2009; Sieber et al., 2010, 2015).

Since the butyryl-CoA dehydrogenase-EtfAB complex contains only FAD as prosthetic groups, it was proposed that the new coupling mechanism was flavin based and was therefore dubbed flavin-based electron bifurcation (Thauer et al., 2008). Since then it has been shown that also the Bcd-EtfAB complexes purified from other butyric-acid- forming anaerobes are electron bifurcating, namely the complexes from *A. fermentans* (Chowdhury et al., 2014), *Clostridium difficile* (Aboulnaga et al., 2013), *Eubacterium limosum* (Jeong et al., 2015) and *M. elsdenii* (Chowdhury et al., 2015). For a crystal structure and the composition of the butyryl-CoA dehydrogenase-EtfAB complex see below.

Based on the results obtained with purified butyryl-CoA dehydrogenase-EtfAB, the earlier finding that the reduction of ferredoxin with NADH in cell extracts of *C. kluyveri* was acetyl-CoA dependent (reaction 1) can now be explained. In cell extracts acetyl-CoA was reduced with NADH via acetoacetyl-CoA and β-hydroxybutyryl-CoA to crotonyl-CoA, which served in addition to NADH as substrate of the butyryl-CoA dehydrogenase-EtfAB complex. The results also explain why several attempts to purify the acetyl-CoA-dependent NADH: ferredoxin reductase activity had not been successful: for activity, four different enzymes or complexes are required, namely β-ketothiolase, β-hydroxybutyryl-CoA dehydrogenase, crotonase and the electron-bifurcating butyryl-CoA dehydrogenase-EtfAB complex.

When we tried to integrate reaction 13 in the energy metabolism of *C. kluyveri* growing on crotonate (Figure 1) or ethanol and acetate (Figure 13 in Buckel and Thauer, 2013) it became apparent that something was still missing. The regeneration of reduced ferredoxin for the reduction of protons to H_2_ could not be explained. This was only possible 2 years later after the electron-bifurcating transhydrogenase NfnAB was discovered (see below).

**Figure 2:**
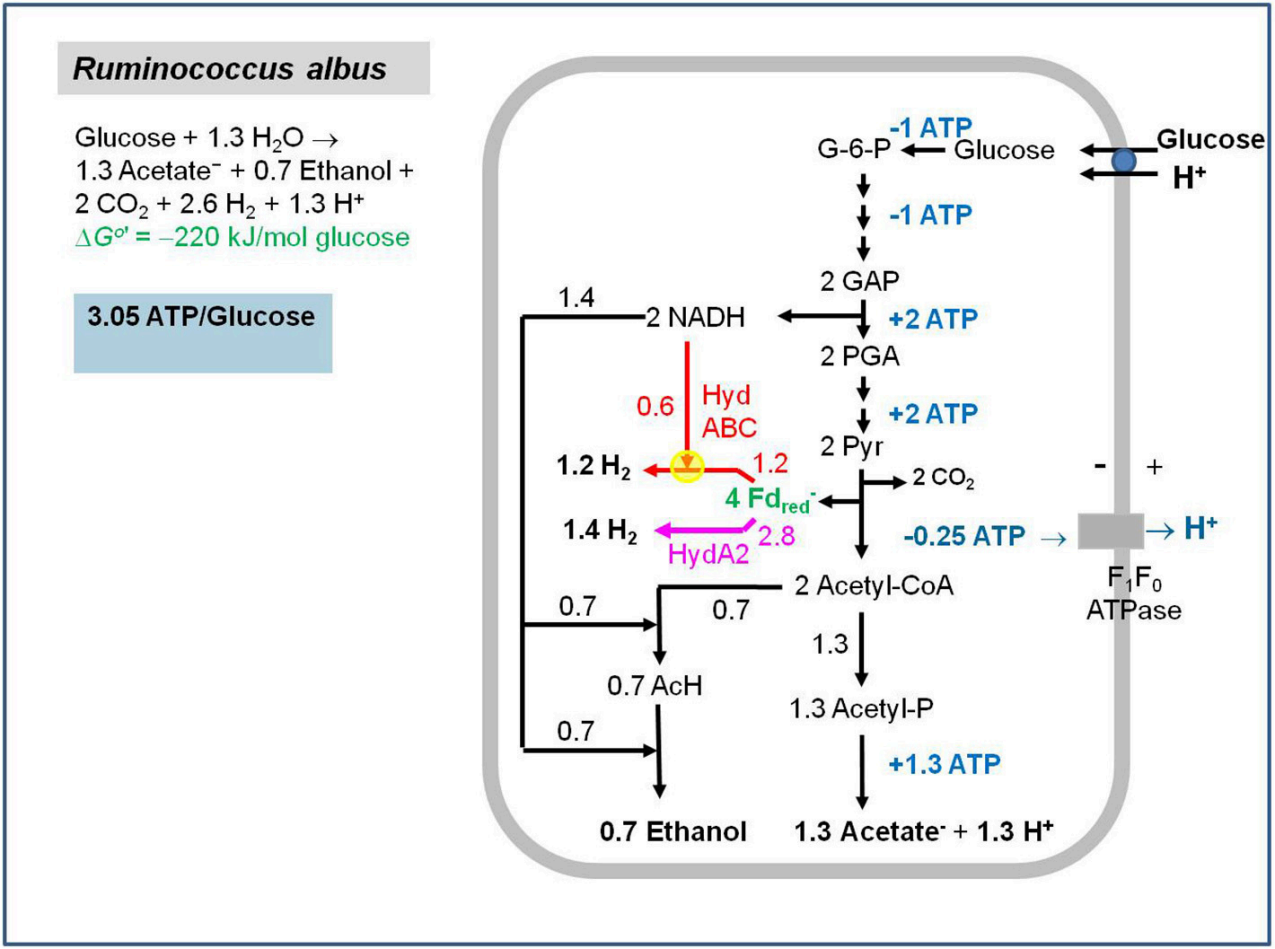
Energy metabolism of *Ruminococcus albus* growing in batch culture on glucose. Glucose used in anabolism is not considered. The yellow dot represents the electron-bifurcating hydrogenase HydABC. The pink arrow represents the non-bifurcating hydrogenase HydA2 (Zheng et al., 2014). G-6-P, glucose-6-phosphate; GAP, glyceraldehyde phosphate; PGA, 1, 3-bisphosphoglycerate; Pyr, pyruvate; Acetyl-P, acetyl-phosphate; AcH, acetaldehyde.

One experimental finding remains to be discussed, namely that cell extracts of *Ruminococcus albus* exhibit acetyl-CoA dependent NADH: ferredoxin reductase activity (Tewes and Thauer, 1980; Zheng et al., 2014) even though it is known that *R. albus* does not form butyrate and genes for the EtfAB-Bcd complex are not found in its genome (Suen et al., 2011). *R. albus* grows on sugars and ferments these via an NAD-specific glyceraldehyde-phosphate dehydrogenase (1,3-bisphosphoglycerate/ glyceraldehyde-3-phosphate: E_0_′ = −310 mV) (Cornell et al., 1979) and a pyruvate: ferredoxin oxidoreductase (acetyl-CoA + CO_2_/pyruvate: E_0_′ = −500 mV) to ethanol, acetate, CO_2_ and H_2_,with more H_2_ formed than can be accounted for by ferredoxin reduction with pyruvate (Thauer et al., 1977). We now know that the rumen bacterium contains an electron-confurcating [FeFe]-hydrogenase (Zheng et al., 2014) (Figure 2) that was first discovered by the group of Mike Adams in *Thermotoga maritima* (Schut and Adams, 2009) (see directly below). The very low activity of acetyl-CoA-dependent NADH: ferredoxin reductase in cell extracts (0.04 μmol per min per mg protein) relative to that of other catabolic enzymes (>1 μmol per min per mg protein) points to an anabolic enzyme. All of this is mentioned here because the acetyl-CoA- dependent NADH: ferredoxin reductase activity in *R. albus* distracted us for some years from connecting the activity with butyrate formation.

## Finding of 10 other electron-bifurcating flavoenzyme complexes

All the findings described below were obtained with purified enzyme complexes following the famous dictum of Efraim Racker “Don't waste clean thinking on dirty enzymes” (Kornberg, 2000). Only electron-bifurcating reactions are considered, for which the stoichiometry was determined in order not to be fooled by indirect effects.

The 10 purified complexes in the following to be discussed all have two things in common: they contain at least one flavin-harboring subunit and they catalyze a ferredoxin/flavodoxin-dependent reaction. Furthermore the 10 electron-bifurcating complexes contain besides flavins also iron-sulfur-clusters and some of them additionally [FeFe]-, [NiFe]-, W-, or Mo-molybdopterin centers. Only butyryl-CoA dehydrogenase-EtfAB contains only flavins (Table 1). The electron-bifurcating complexes are generally located in the cytoplasm. An exception is the ubiquinone reductase (FixCX)-FixAB complex, of which FixC is integrated into the membrane and FixAB is homologous to EtfBA. In the case of the EtfAB-containing complexes and NfnAB, which contain several FAD, the electron-bifurcating flavin has been identified. Most of the other complexes contain only one flavin, either FMN or FAD. It remains to be shown that those flavins are indeed the centers of electron bifurcation.

In the electron-bifurcating flavoproteins analyzed to date, the putative electron-bifurcating flavin is relatively loosely bound, when in the reduced state. Therefore, FAD or FMN had to be added to the reducing buffers during anoxic purification, otherwise the purified enzyme complexes were inactive. *Vice versa*, EtfAB from *A. fermentans* or *M. elsdenii*, which can be purified in the oxidized state under air, loses the α-FAD, whereas the bifurcating β-FAD remains tightly bound to the protein (Sato et al., 2013; Chowdhury et al., 2014). However, the activities could at least be partially restored upon addition of FAD.

### 2009: finding of electron-bifurcating, NAD-specific [FeFe]-hydrogenase HydABC

The group of Mike Adams in Athens, Georgia, USA (Schut and Adams, 2009) isolated an FMN-containing cytoplasmic [FeFe]-hydrogenase complex (HydABC) from *T. maritima* that ferments glucose to 2 acetate, 2 CO_2_ and 4 H_2_ involving an NAD-specific GAPDH (1,3-bisphosphoglycerate/glyceraldehyde-3-phosphate: E_0_′ = −310 mV) (Cornell et al., 1979) and a pyruvate: ferredoxin oxidoreductase (acetyl-CoA + CO_2_/pyruvate: E_0_′ = −500 mV); for a metabolic scheme see Figure 12 in Buckel and Thauer (2013). The enzyme complex stoichiometrically couples the endergonic formation of H_2_ (E_0_′ = −414 mV) from NADH (E_0_′ = −320 mV) to the exergonic formation of H_2_ from reduced ferredoxin (E_0_′ = −420 mV) (reaction 14). HydA carries the active site [FeFe]-center (H-cluster) and shows sequence similarity to the one-subunit non-bifurcating [FeFe]-hydrogenase from *C. pasteurianum*. HydB has sequence similarity to NuoF, the FMN-harboring subunit of the NADH dehydrogenase complex in the *E. coli* respiratory chain. HydC is an iron-sulfur protein. When purified in the presence of FMN, the active preparation contained 1 FMN per HydABC: the flavin is most probably associated with HydB and is required there as a two-electron/one-electron switch for electron transport from NADH via iron-sulfur-clusters to ferredoxin and H^+^.

(14) 2 ${\text{Fd}}_{\text{red}}^{-}$ + NADH + 3 H^+^ ⇋ 2 Fd_ox_ + NAD^+^ + 2 H_2_
  ΔG°′ near +17 kJ/mol

**Figure 3:**
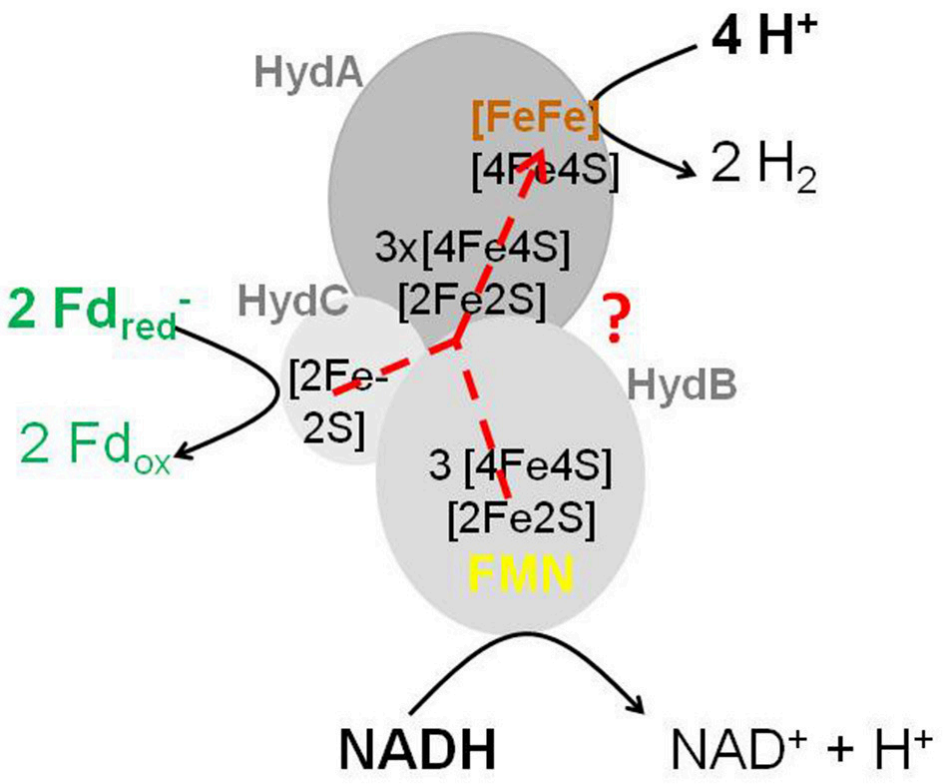
Scheme of the electron-bifurcating [FeFe]-hydrogenase HydABC. The electron-bifurcating hydrogenase from *Acetobacterium woodii* contains a fourth subunit without a prosthetic group (Poehlein et al., 2012; Schuchmann and Müller, 2012). As drawn, the FMN in HydB cannot be the site of electron bifurcation The presence of a second flavin was proposed (Buckel and Thauer, 2013).

Under physiological conditions the free energy change associated with reaction 14 is near 0 kJ/mol because *in vivo* the reduction potential E' of the Fd_ox_/Fd_red_ couple is probably close to that of the acetyl-CoA + CO_2_/pyruvate couple (E_0_′ = −500 mV). The scheme of the HydABC complex that catalyzes reaction 14 (Figure 3) highlights that the FMN in HydB cannot be the electron-confurcating/bifurcating flavin. A crystal structure of the active enzyme soaked with FMN is urgently needed to find out whether a second FMN is bound to the complex.

The electron-confurcating/bifurcating enzyme complex is also present in many other H_2_-forming bacteria such as *R. albus* (Figure 2) (Zheng et al., 2014) and *S. wolfei* (Sieber et al., 2015) and in many hydrogenotrophic acetogens, such as *A. woodii* (Poehlein et al., 2012; Schuchmann and Müller, 2012) and *Moorella thermoacetica* (Wang et al., 2013b). In the acetogens growing on H_2_ and CO_2_, the enzyme complex catalyzes the reverse, electron-bifurcating reaction. For reviews on [FeFe]-hydrogenases see Peters et al. (2015) and Sondergaard et al. (2016).

### 2010: finding of electron-bifurcating transhydrogenase NfnAB

After 40 years, the group of Rudolf Thauer (Wang et al., 2010) again attempted to purify the enzyme system that catalyzes reaction 2 in cell extracts of *C. kluyveri*. The cell extracts also catalyzed an NADH-dependent reduction of NADP^+^ with reduced ferredoxin (Thauer et al., 1971), which indicated the presence of a ferredoxin: NADP reductase. The breakthrough was the finding in the genome of *C. kluyveri* (Seedorf et al., 2008) of two adjacent genes, the product of one of which showed sequence similarity to ferredoxin: NADP reductase from plants, which has transhydrogenase side activity (Böger, 1971). Furthermore, the genes are located within the gene cluster for acetoacetyl-CoA reduction to butyryl-CoA. Heterologous expression of the genes, together or individually, finally led to the purified active His_6_-tagged enzyme complex catalyzing the reversible reaction 15 (The crystal structure and composition are discussed in a separate section). Purification of NfnAB from cell extracts of *C. kluyveri* failed because during the chromatographic steps the two subunits separated. The cytoplasmic FAD-containing transhydrogenase complex was abbreviated as NfnAB for **N**ADH-dependent **f**erredoxin: **N**ADP^+^ reductase (Wang et al., 2010).

(15) 2 ${\text{Fd}}_{\text{red}}^{-}$ + NADH + 2 NADP^+^ + H^+^ ⇋ 2 Fd_ox_ + NAD^+^ + 2 NADPH
  ΔG°′ near −20 kJ/mol

Under standard conditions (Fd_ox_/Fd_red_; E_0_′ = −420 mV; NAD(P)^+^/NAD(P)H: E_0_′ = −320 mV) the free energy change associated with reaction 15 is exergonic by −20 kJ/mol. Under physiological conditions the free energy change is near 0 kJ/mol because the NADH/NAD^+^ ratio in the cells is below 1 and the NADPH/NADP^+^ ratio above 1. How much below and above is not really known because the *in vivo* ratios are very difficult to determine experimentally, especially for strict anaerobes (Decker and Pfitzer, 1972; Snoep et al., 1991; Beri et al., 2016). But what is definitively known is that in aerobes the transhydrogenation from NADH to NADP^+^ requires energy whereas the reverse reaction does not (Sauer et al., 2004). In aerobically growing *E. coli* the reduction potentials of the NAD^+^/NADH couple and of the NADP^+^/NADPH couple are probably up to 100 mV apart as estimated from the energy available for the transhydrogenation reaction (Sauer et al., 2004) and from the ratios determined experimentally (Bennett et al., 2009). In aerobically growing *E. coli* the NADH/NAD^+^ ratio appears to be lower than in anaerobically growing cells (Wimpenny and Firth, 1972; de Graef et al., 1999; Nikel et al., 2008). However, in anaerobically growing *E. coli* the NADH/NAD^+^ ratio can be lowered to that found in aerobically growing cells when the biosynthesis of NAD is increased (Liang et al., 2012). Prediction is that NADPH/NADP^+^ ratios are much higher than 1 as estimated from the free energy changes associated with most NADPH-regenerating systems (Spaans et al., 2015).

In the energy metabolism of *C. kluyveri* growing on crotonate (reaction 5), NfnAB functions in transferring electrons from NADPH to both NAD^+^ and ferredoxin (Figure 1). NADP^+^ reduction via β-hydroxybutyryl-CoA dehydrogenation is driven by the exergonic cleavage of acetoacetyl-CoA to 2 acetyl-CoA (ΔG°′ = −25 kJ/mol) and NADH oxidation is driven by the exergonic reduction of crotonyl-CoA (ΔG°′ = −40 kJ/mol) (reaction 13). When *C. kluyveri* grows on ethanol and acetate the function of NfnAB is to catalyze the formation of NADPH; see Figure 12 in Buckel and Thauer (2013).

The genes for the NfnAB complex are widely distributed among anaerobic bacteria and archaea (Wang et al., 2010; Huang et al., 2012; Demmer et al., 2015; Nguyen et al., 2017). In the genome of *C. ljungdahlii* the two genes are fused (Köpke et al., 2010). In the genome of *P. furiosus* there are two sets of NfnAB genes, both of which are expressed (Nguyen et al., 2017). Nfn1, which catalyzes reaction 15, has been purified and characterized and a crystal structure was obtained (Lubner et al., 2017). Besides reaction 15, Nfn1 also catalyzes the reduction of polysulfide with NADPH rather than with NADH; Nfn1 is identical with NADP-specific sulfide dehydrogenase purified 23 years ago by Ma and Adams (1994). Nfn2 shows ferredoxin: NADP oxidoreductase activity and appears not to be electron bifurcating (Nguyen et al., 2017). In some anaerobes, e.g., in *C. pasteurianum* and *A. woodii*, the *nfnAB* genes are lacking.

In 1972, in the lab of Harland Wood at Case Western Reserve, Cleveland, Ohio, USA, Rudolf Thauer studied the reduction of CO_2_ to formate in cell extracts of *M. thermoacetica* (formerly *Clostridium thermoaceticum*) (Thauer, 1972). It had previously been shown that formate oxidation in cell extracts is NADP^+^ dependent (Li et al., 1966). Therefore, it was predicted that CO_2_ reduction to formate requires NADPH, but this was difficult to show, because *M. thermoacetica* is a thermophile. The use of glucose-6-phosphate and glucose-6-phosphate dehydrogenase from yeast to regenerate NADPH failed because of thermo-inactivation of the dehydrogenase. However, NADPH could be regenerated with H_2_ after addition of ferredoxin and heat-stable hydrogenase from *C. pasteurianum* to the cell extracts. Surprisingly, NADPH regeneration and CO_2_ reduction with H_2_ was dependent on the addition of NADH (Thauer, 1972). These results can now be understood knowing that cell extracts of *M. thermoacetica* contain Nfn (Wang et al., 2013b).

### 2011: finding of electron-bifurcating heterodisulfide reductase HdrABC-MvhADG

In 1977 Robert Gunsalus and Ralph Wolfe reported that cell extracts of *Methanobacterium thermoautotrophicum* (renamed *Methanothermobacter thermoautotrophicus*) catalyzed the reduction of CO_2_ to methane only in the presence of catalytic amounts of methyl-coenzyme M, which was itself reduced to methane (Gunsalus and Wolfe, 1977). They interpreted this finding, from then on dubbed the RPG effect (RPG for R. P. Gunsalus), to indicate that the first, CO_2_-reducing step and the last, methyl-coenzyme M-reducing step were somehow coupled. Since the membrane fraction was not required, a chemiosmotic coupling was not very likely. It took over 30 years to show that the two reactions were coupled via oxidation (first step) and reduction (last step) of ferredoxin; see the commentary by Thauer (2012).

In 2008, after a seminar talk of Reiner Hedderich in Marburg on the reduction of CoM-S-S-CoB with H_2_ catalyzed by the MvhADG-HdrABC complex from *Methanothermobacter marburgensis* (Hedderich et al., 2005), Wolfgang Buckel raised the question whether the complex, like the butyryl-CoA dehydrogenase-EtfAB complex in *C. kluyveri*, could be electron bifurcating. The reason behind this question was that the complex contained FAD (in subunit HdrA) of unknown function (Hedderich et al., 1994; Künkel et al., 1997) and it had a too low specific activity when tested with H_2_ and CoM-S-S-CoM (Setzke et al., 1994). This discussion led Anne Kaster in the group of Rudolf Thauer to test whether the reaction catalyzed by MvhADG-HdrABC is ferredoxin dependent, which she found to be the case. Upon addition of ferredoxin, the specific activity of heterodisulfide reduction with H_2_ increased over 100-fold. We therefore proposed that the complex couples the endergonic reduction of ferredoxin (E_0_′ = −420 mV) with H_2_ (E_0_′ = −414 mV) to the exergonic reduction of CoM-S-S-CoB (E_0_′ = −140 mV) with H_2_ (reaction 16) (Thauer et al., 2008).

(16) 2 H_2_ + CoM-S-S-CoB + 2 Fd_ox_ → HS-CoM + HS-CoB + 2 ${\text{Fd}}_{\text{red}}^{-}$ + 2 H^+^
  ΔG°′ near −52 kJ/mol

Finally, in 2011 the proposed stoichiometry was experimentally confirmed (Kaster et al., 2011). It took 3 years because the MvhADG-HdrABC complex (for crystal structure and subunit functions, see below) is extremely oxygen sensitive and prone to uncoupling and because CoM-S-S-CoB disproportionates to CoM-S-S-CoM and CoB-S-S-CoB under most analytical conditions (Kaster et al., 2011). There is also evidence for uncoupling *in vivo*. The growth yield of *M. marburgensis* decreases with increasing the supply of H_2_ and CO_2_ to exponentially growing batch cultures (Schönheit et al., 1980; Morgan et al., 1997).

Many hydrogenotrophic methanogens can also grow on formate (E_0_′ = −430 mV) as sole energy source. In 2010 the group of John Leigh at the University of Washington, Seattle, WA, USA, showed that in cells grown on formate HdrABC forms a complex with formate dehydrogenase (FdhAB) (Wood et al., 2003); they proposed that the complex catalyze reaction 17 (Costa et al., 2010).

(17) 2 HCOO^−^ + CoM-S-S-CoB + 2 Fd_ox_ → CO_2_ + HS-CoM + HS-CoB + 2 ${\text{Fd}}_{\text{red}}^{-}$
  ΔG°′ near −58 kJ/mol

The gene encoding the flavin-harboring HdrA subunit is not only found in methanogenic and methanotrophic archaea (Costa et al., 2013; Costa and Leigh, 2014; Lang et al., 2015; Browne et al., 2017; Yan et al., 2017) but also in sulfate reducing anaerobic bacteria and archaea (Grein et al., 2013; Meyer et al., 2013, 2014; Price et al., 2014; Ramos et al., 2015; Otwell et al., 2016), sulfur-oxidizing bacteria (Mangold et al., 2011; Grein et al., 2013; Liu et al., 2013), strict anaerobes having the capacity to degrade aromatic compounds (Boll et al., 2014, 2016), *Syntrophorhabdus aromaticivorans* (Nobu et al., 2015) and some acetogenic bacteria (Mock et al., 2014).

### 2013: finding of electron-bifurcating caffeyl-CoA reductase CarCDE

Johannes Bertsch in the group of Volker Müller in Frankfurt, Germany, isolated from *A. woodii* grown on H_2_, CO_2_ and caffeate a cytoplasmic FAD- containing caffeyl-CoA reductase-EtfAB complex encoded by the genes *carC* (caffeyl-CoA reductase)*, cardD* (EtfB) and *carE* (EtfA) respectively, that stoichiometrically couples the endergonic reduction of ferredoxin with NADH to the exergonic reduction of caffeyl-CoA to dihydrocaffeyl-CoA with NADH (reaction 18) (Bertsch et al., 2013). Reaction 18 is analogous to reaction 13. (E_0_′ of the dihydrocaffeyl-CoA/caffeyl-CoA couple estimated to be −30 mV) (Buckel and Thauer, in press).

(18) 2 NADH + Caffeyl-CoA + 2 Fd_ox_ → 2 NAD^+^ + Dihydrocaffeyl-CoA + 2 ${\text{Fd}}_{\text{red}}^{-}$ + 2 H^+^
  ΔG°′ near −36 kJ/mol

The caffeyl-CoA reductase-EtfAB complex contains three FAD (one per subunit) and two [4Fe-4S]-clusters (Table 1) as deduced from a ferredoxin-like sequence with eight cysteines at the N-terminus of CarE and confirmed by iron- and acid-labile sulfur determinations (Bertsch et al., 2013).

Growth on H_2_, CO_2_ and caffeate is possible because *A. woodii* also harbors an active electron-bifurcating hydrogenase (reaction 14) (Schuchmann and Müller, 2012), a sodium ion-translocating Rnf complex (Müller et al., 2008; Biegel and Müller, 2010) (reaction 12) and a sodium ion-translocating ATP synthase complex (Brandt et al., 2016) (reaction 9), all of which were characterized by the group of Volker Müller (Müller, 2003).

### 2013: finding of electron-bifurcating, NADP-specific [FeFe]-hydrogenase HytA-E

Shuning Wang in the group of Rudolf Thauer (Wang S. N. et al., 2013) characterized from *Clostridium autoethanogenum* a cytoplasmic FMN-containing [FeFe]-hydrogenase complex (HytA-E) that stoichiometrically couples the endergonic reduction of ferredoxin with H_2_ to the exergonic reduction of NADP^+^ with H_2_ (reaction 19). (The **t** in Hyt stands for TPN, the old name for NADP). The amino acid sequence of HytB is similar to that of NuoF, the FMN-harboring subunit of the NADH dehydrogenase complex in the *E. coli* respiratory chain. For the function of the complex in *C. autoethanogenum* fermenting H_2_ and CO_2_ to ethanol see Mock et al. (2015). Reaction 19 is analogous to reaction 14.

(19) 2 Fd_ox_ + NADP^+^ + 2 H_2_ ⇋ 2 ${\text{Fd}}_{\text{red}}^{-}$ + NADPH + 3 H^+^
  ΔG°′ near −17 kJ/mol

### 2013: finding of electron-bifurcating formate dehydrogenase FdhF2-HylABC

Also in 2013, Wang et al. characterized from *Clostridium acidiurici* a cytoplasmic FMN-containing formate dehydrogenase complex (HylABC-FdhF2) (Hyl for **hy**drogenase **l**ike) that reversibly couples the endergonic reduction of ferredoxin with formate to the exergonic reduction of NAD^+^ with formate (E_0_′ = −430 mV) in the stoichiometry given in reaction 20 (Wang et al., 2013a). HylB has sequence similarity to NuoF, the FMN-harboring subunit of the NADH dehydrogenase complex in the *E. coli* respiratory chain.

(20) 2 HCOO^−^ + 2 Fd_ox_ + NAD^+^ + ⇋ 2 CO_2_ + 2 ${\text{Fd}}_{\text{red}}^{-}$ + NADH + H^+^
  ΔG°′ near −24 kJ/mol NAD

The dependence of the formate dehydrogenase on reduced ferredoxin and NAD^+^ had already been observed by Rudolf Thauer in 1973 in the laboratory of Harland Wood. In cell extracts of *C. acidi-urici*, CO_2_ was only reduced to formate when both reduced ferredoxin and NADH were continuously regenerated (Thauer, 1973).

### 2014: finding of electron-bifurcating methylene-H_4_F reductase MetFV-HdrABCMvhD

Johanna Mock in the group of Rudolf Thauer purified a MetFV-HdrABCMvhD complex from *M. thermoacetica* that catalyzes in the Wood-Ljungdahl pathway the reduction of methylene-tetrahydrofolate (methylene-H_4_F) to methyl-tetrahydrofolate (methyl-H_4_F) (E_0_′ = −200 mV) (Wohlfarth and Diekert, 1991) with reduced benzyl viologen (E_0_′ = −360 mV) and the reduction of benzyl viologen with NADH (Mock et al., 2014). From their results they proposed that the FAD- and FMN-containing complex couples the endergonic reduction of a yet to be identified specific ferredoxin/flavodoxin with NADH to the exergonic reduction of methylene-H_4_F with NADH (reaction 21). They showed that subunit HdrA harbors two FAD, and proposed that one FAD is the electron-bifurcating flavin.

(21) 2 NADH + Methylene-H_4_F + Fd_ox_(?) ⇋ 2 NAD^+^ + Methyl-H_4_F + 2 ${\text{Fd}}_{\text{red}}^{-}$ (?)
  ΔG°′ near −5 kJ/mol

Notably, the methylene-H_4_F reductases from *Blautia producta* (formerly *Peptostreptococcus productus*) (Wohlfarth et al., 1990) and from *A. woodii* (Bertsch et al., 2015) are not electron bifurcating, which indicates that not all acetogenic bacteria can couple this reaction with the reduction of ferredoxin.

### 2015: finding of electron-bifurcating lactate dehydrogenase LctBCD

Marie Weghoff in the group of Volker Müller (Weghoff et al., 2015) discovered in *A. woodii* a cytoplasmic FAD-containing lactate dehydrogenase-EtfAB complex (Table 1) encoded by the genes *lctD* (lactate dehydrogenase), *lctC* (EtfA) and *lctB* (EtfB), respectively, that reversibly couples the endergonic reduction of NAD^+^ (E_0_′ = −320 mV) with lactate (E_0_′ = −190 mV) to the exergonic reduction of NAD^+^ with reduced ferredoxin (E_0_′ = −420 mV) (reaction 22). The enzyme complex appears to be present in all anaerobic microorganisms that can ferment lactate without containing an FAD-dependent lactate dehydrogenase or a membrane-associated lactate dehydrogenase that couples lactate oxidation to the reduction of menaquinone (Garvie, 1980; Thomas et al., 2011).

(22) Lactate^−^ + 2 NAD^+^ + 2 ${\text{Fd}}_{\text{red}}^{-}$ ⇋ Pyruvate^−^ + 2 NADH + 2 Fd_ox_
  ΔG°′ near +6 kJ/mol

A crystal structure for the lactate dehydrogenase-EtfAB complex is not yet available. The primary structure predicts the complex to contain one FAD per subunit LctD, LctC and LctB and one [4Fe-4S]-cluster in LctC (Table 1) (Weghoff et al., 2015).

Over 40 years ago lactate dehydrogenase of *M. elsdenii* was reported to transfer electrons under aerobic conditions from lactate to EtfAB, from where the electrons could be transferred to crotonyl-CoA in the presence of butyryl-CoA dehydrogenase (Brockman and Wood, 1975). An electron transfer from lactate via EtfAB to NAD was not observed, for which—as we now know—reduced ferredoxin would have been required.

Mammals contain only NAD-specific lactate dehydrogenases. In their cells the thermodynamic problem is solved by coupling lactate oxidation to pyruvate with NAD^+^ to an energy-dependent transport of pyruvate and NADH into the mitochondria (Hashimoto et al., 2006; Jacobs et al., 2013).

### 2017: finding of electron-bifurcating, F_420_-specific heterodisulfide reductase HdrA2B2C2

James Ferry‘s group at Penn State, USA, showed that the cytoplasmic FAD-containing HdrA2B2C2 complex from *Methanosarcina acetivorans* couples the endergonic reduction of ferredoxin (E_0_′ = −420 mV) with F_420_H_2_ (E_0_′ = −360 mV) (Walsh, 1986) to the exergonic reduction of the heterodisulfide CoM-S-S-CoB (E_0_′ = −140 mV) (Tietze et al., 2003) with F_420_H_2_ (reaction 23) (Yan et al., 2017).

(23) 2 F_420_H_2_ + CoM-S-S-CoB + 2 Fd_ox_ → 2 F_420_ + HS-CoM + HS-CoB + 2 ${\text{Fd}}_{\text{red}}^{-}$ + 2 H^+^
  ΔG°′ near −30 kJ/mol

The function of the complex *in vivo* remains to be elucidated. It is not yet known, in which reaction the reduced F_420_ is regenerated. One possibility is regeneration via F_420_ reduction with ferredoxin. The cells of *M*. acetivorans contain an Rnf homolog that could mediate F_420_ reduction with reduced ferredoxin regenerated in the CO dehydrogenase reaction, although this has not yet been shown (Welte and Deppenmeier, 2014).

### 2017: finding of electron-bifurcating, NAD-specific ubiquinol reductase FixABCX

In 2004, based on genetic studies, Stefan Nordlund from the University of Stockholm, Sweden, proposed that the *fixABCX* genes in *R. rubrum* encode a membrane-bound enzyme complex that plays a central role in electron transfer to nitrogenase (Edgren and Nordlund, 2004). Two years later they published evidence that a specific ferredoxin (FixN) mediates electron transfer from the FixABCX complex to nitrogenase (Edgren and Nordlund, 2005). In 2008 Wolfgang Buckel proposed that FixABCX bifurcates the two electrons from NADH (Herrmann et al., 2008). One electron should go to the respiratory chain and the other reduces ferredoxin, because FixA is homologous to EtfB and FixB homologous to EtfA.

Finally, in 2017 a group headed by Lance Seefeldt in Logan, Utah, USA, provided *in vitro* evidence that the membrane-associated FixABCX complex from *A. vinelandii* couples the endergonic reduction of flavodoxin semiquinone (Fld_sq_) (E_0_′ = −420 mV) with NADH (E_0_′ = −320 mV) to the exergonic reduction of ubiquinone (Q) (E_0_′ = +90 mV) with NADH (reaction 24) (Ledbetter et al., 2017). FixC is a membrane-associated flavoprotein with ubiquinone reductase activity. Attached to FixC are FixAB and the iron-sulfur protein FixX that probably transfers electrons from the electron-bifurcating FixA to the ferredoxin FixN or the flavodoxin semiquinone yielding the flavodoxin hydroquinone (Fld_hq_). Evidence was also provided that the electron-bifurcating complex is operative *in vivo*, when *A. vinelandii* grows aerobically under N_2_-fixing conditions (Ledbetter et al., 2017). Thus flavin-based electron bifurcation is not restricted to anaerobic microorganisms as previously thought.

(24) 2 NADH + Q + 2 Fld_sq_ → 2 NAD^+^ + QH_2_ + 2 ${\text{Fld}}_{\text{hq}}^{-}$
  ΔG°′ near −60 kJ/mol

## Crystal structure of four electron-bifurcating flavoenzyme complexes

As basis for a mechanistic understanding, the crystal structures of EtfAB, the butyryl-CoA dehydrogenase-EtfAB complex (Bcd_2_-EtfAB)_4_ (reaction 13), the caffeyl-CoA reductase complex (CarCDE)_4_ **(**reaction 18), the NfnAB complex (reaction 15), and the MvhADG-HdrABC complex (reaction 16) have in the meantime been obtained.

In 2014 the crystal structure of recombinant electron-bifurcating **EtfAB** from *A. fermentans* was solved in the group of Wolfgang Buckel in collaboration with Ulrich Ermler in Frankfurt, Germany (Chowdhury et al., 2014). Like the non-bifurcating Etfs involved in β-oxidation of fatty acids, *A. fermentans* EtfAB is composed of three domains; subunit A consists of domains I + II, and subunit B of domain III. Domains I and III form the rigid part of the complex, whereas the a-FAD containing domain II can rotate up to 90°. Domain III contains the bifurcating b-FAD, which is replaced in the non-bifurcating Etfs by AMP. b-FAD was shown to be the site of NADH- and ferredoxin binding; with 18 Å apart, it is not in electron-transfer distance to a-FAD in domain II. However, only a rotation of domain II by10° reduces the distance to only 14 Å. This state, which allows electron transfer from b-FAD to a-FAD, has been called the bifurcation or B-state.

In 2017, the structure of **(Bcd-EtfAB)**_**4**_ **complex** from *C. difficile* was finally obtained (Demmer et al., 2017). In contrast to the EtfAB structure, domain II of EtfA has rotated by 80°, which brought a-FAD only 8 Å apart from the d-FAD in Bcd. This state, which allows rapid electron transfer from a-FAD to d-FAD, has been called the dehydrogenase or D-state. Electron bifurcation is predicted to start with binding of NADH near b-FAD, which induces a rotation or swing of domain II back to the B-state. Hydride transfer from NADH reduces b-FAD to b-FADH^−^, which bifurcates. One electron goes to the high potential a-FAD, which swings by 90° rotation of domain II to Bcd and transfers one electron to d-FAD. The remaining “hot” semiquinone of b-FAD immediately “shoots” an electron to the nearby low potential ferredoxin (6 Å apart). Repetition of this process reduces another ferredoxin and donates a second electron to d-FAD forming d-FADH^−^, which converts crotonyl-CoA to butyryl-CoA (Chowdhury et al., 2016; Demmer et al., 2017; Buckel and Thauer, in press).

Beginning 2018, the 3.5 Å structure of the (**CarCDE**)_**4**_ **complex** from *A. woodii* was published that catalyzes the ferredoxin-dependent reduction of caffeyl-CoA to dihydrocaffeyl-CoA (Demmer et al., 2018). CarE, homologous to EtfA, contains an additional ferredoxin-like domain with two [4Fe-4S] clusters N-terminally fused. “It might serve, *in vivo*, as specific adaptor for the physiological electron acceptor. Kinetic analysis of a CarCDE(ΔFd) complex indicates the bypassing of the ferredoxin-like domain by artificial electron acceptors. Site-directed mutagenesis studies substantiated the crucial role of the C-terminal arm of CarD and of ArgE203, hydrogen-bonded to the bifurcating FAD, for flavin based electron bifurcation” (Demmer et al., 2018).

In 2015 the group of Ulrich Ermler (Demmer et al., 2015, 2016) solved the crystal structure of the heterologously produced electron-bifurcating **NfnAB complex** (reaction 15) from *T. maritima*. NfnB harbors one FAD, a proximal [4Fe-4S]-cluster and a distal [4Fe-4S]-cluster, whereas NfnA contains one [2Fe-2S]-cluster and one FAD. NADPH binds near the FAD in NfnB, NAD near the FAD in NfnA and ferredoxin most likely near the distal [4Fe-4S]-cluster in NfnB. The overall structure indicates that FAD in NfnB is the site of electron bifurcation. One electron from NADPH is predicted to be transferred from the FAD in NfnB to the [2Fe-2S]-cluster and further via the FAD in NfnA to NAD^+^. The other electron probably jumps from FAD in NfnB via the proximal and the distal [4Fe-4S]-clusters to ferredoxin. Only the FAD in NfnB and the [2Fe-2S]-cluster are not in electron-transfer distance (15 Å apart). However, electron transfer could become possible by a small conformational change of 2–3 Å without disturbing the tertiary structure. The crystal structure of NfnAB from *P. furiosus* recently confirmed this picture (Lubner et al., 2017; Berry et al., 2018).

In 2017 Seigo Shima's group in Marburg solved together with Ulrich Ermler in Frankfurt the crystal structure of the electron-bifurcating **MvhADG-HdrABC complex** (reaction16), which they isolated from *Methanothermococcus thermolitotrophicus* and crystallized under strictly anoxic conditions (Wagner et al., 2017). They showed that the subunit HdrA harbors one FAD, the only flavin present in the complex, six partially unusual [4Fe-4S]-clusters and the ferredoxin binding site. HdrB contains two novel non-cubane [4Fe-4S]-clusters between which the heterodisulfide binds and is reduced. HdrC contains two [4Fe-4S]-clusters and is positioned for transport of electrons between HdrA and HdrB. MvhA contains the [NiFe]-hydrogenase active site, MvhG contains three [4Fe-4S]-clusters and MvhD contains one [2Fe-2S]-cluster; the latter is in electron-transfer distance to one of the [4Fe-4S]-clusters in HdrA. The authors discuss several possibilities of how FAD in HdrA could bifurcate the electrons on their way from H_2_ to the heterodisulfide and ferredoxin; for a commentary see Lubner and Peters (2017).

Interestingly, the high-resolution crystal structure of MvhADG-HdrABC revealed that the active site of HdrB lacks zinc; based on EXAFS data it had been proposed that zinc is involved in heterodisulfide reduction (Hamann et al., 2007, 2009). Most likely, the zinc replaced one of the iron in the non-cubane iron-sulfur-clusters of the active site. In the EXAFS studies it had not been checked before analysis, whether the complex still had full heterodisulfide reductase activity, which is rapidly lost upon storage.

## Proposed mechanism of flavin-based electron bifurcation

In 2012, Wolfgang Nitschke and Michael Russel proposed a catalytic mechanism based on the assumption that in electron-bifurcating flavoproteins the bound flavin has similar redox properties as free flavins. The inherent property of free flavins (FAD and FMN) is to have three oxidation states, the ox = 0, quinone state (Q), the ox = −1, semiquinone state (SQ) and the ox = −2, the hydroquinone state (HQ), with crossed-over one-electron reduction potentials: the Q/SQ couple has a more negative redox potential than the SQ/HQ couple. Upon one-electron reduction of Q, the SQ state does not accumulate and can therefore be considered as sort of a transition state. This redox property is usually only observed in the conjugated molecular orbitals of cyclic organics such as benzoquinones and flavins (Nitschke and Russell, 2012; Schoepp-Cothenet et al., 2013). The reduction potential of the Q/SQ couple of flavins in water at pH 7 was determined by pulse radiolysis to be −314 mV. However, when the first electron has a lower potential than the second one, the initially formed SQ does not accumulate because the reduction of one SQ by a second SQ to HQ and Q (disproportionation) is thermodynamically favored. E_0_′ of the Q/HQ couple was determined by normal redox titration to be −219 mV. From E_0_′ of the Q/SQ couple = −314 mV and of the Q/HQ couple = −219 mV, the reduction potential of the SQ/HQ couple was calculated to be −124 mV (Anderson, 1983). The two reduction potentials are thus almost 200 mV apart and confurcate in the reduction potential of the Q/HQ couple. Conversely, upon one-electron oxidation of fully reduced flavins, the electron to leave first is the one with the more positive reduction potential, thereby generating the thermodynamically unstable semiquinone radical with a “hot” electron capable in the case of electron-bifurcating flavoproteins to reduce ferredoxin or flavodoxin (Nitschke and Russell, 2012).

With respect to redox properties, ubiquinone, plastoquinone, and menaquinone are similar to flavins in that they also have crossed-over redox potentials (Takamiya and Dutton, 1979; Kishi et al., 2017), however at least 200 mV more positive than those of free flavins (Bergdoll et al., 2016). In fact, the mechanism proposed above for flavin-based electron bifurcation is derived from the mechanism of benzoquinone-based electron bifurcations in the cytochrome *bc*_1_ complex of the respiratory chain and the cytochromes *b*_6_*f*-complex in oxygenic photosynthesis (Nitschke and Russell, 2012; Bergdoll et al., 2016; Buckel and Thauer, in press).

In 2017, a group headed by John Peters in Bozeman, Montana, USA (Hoben et al., 2017; Lubner et al., 2017; Zhang et al., 2017) provided first experimental evidence that the mechanism of ubiquinone-based electron bifurcation indeed also applies to flavin-based electron bifurcation. For NfnAB they showed that, as predicted, the semiquinone state of the electron-bifurcating FAD in NfnB has a very short lifetime. Whereas in free flavins the crossed-over midpoint reduction potentials are separated by 200 mV (Anderson, 1983), those in FAD bound to NfnB were calculated from stability constants of the flavin SQ to be 1,300 mV apart. The midpoint potential of the flavin Q/HQ couple was determined to be near −300 mV, that of the Q/SQ couple was calculated from the stability constant to be near −950 mV and that of the SQ/HQ couple calculated to be near +350 mV. From −950 mV, the electron can easily flow via the two [4Fe-4S]-clusters to the low reduction potential electron acceptor ferredoxin or flavodoxin. However, the calculated midpoint reduction potential of the flavin SQ/HQ couple of near +350 mV poses the problem that from there the electron has to reach the high potential electron acceptor NAD^+^ (E_0_′ = −320 mV). In between there is an unusual [2Fe-2S]-cluster, whose reduction potential has been determined to be E_0_′ = +80 mV (Hagen et al., 2000). Therefore, under standard conditions the electron transport from the flavin SQ via the [2Fe-2S]-cluster to NAD^+^ is highly endergonic. It should be noted, however, that the one-electron midpoint potential (Q/SQ) of the bifurcating FAD is based on photon excited transient absorption spectroscopy and calculations with partially assumed parameters. Therefore, the derived value certainly does not represent the Q/SQ potential of the ground state, which should be at least 300 mV more positive than −950 mV. As consequence the SQ/HQ potential should be at least 300 mV more negative than +350 mV (Buckel and Thauer, in press).

The electron-bifurcating reactions catalyzed by NfnAB and by the NuoF homologs-containing complexes (Table 1) can function *in vivo* in both directions. The reversibility has been studied in more detail with NfnAB. *In vitro*, the NfnAB- catalyzed reaction 15 was shown to run into equilibrium from both sides and the equilibrium was stable for at least 10 min (Wang et al., 2010). Thus the coupling of the endergonic to the exergonic reaction must have proceeded with 100% efficiency, absolutely without any slips that would have uncoupled the reactions. Interestingly, ubiquinol-based electron bifurcation proceeds also reversibly as evidenced by the involvement of a cytochrome *bc*_1_ complex homolog in reversed electron transport in nitrite oxidizing bacteria; see example given (Lucker et al., 2013).

Noteworthy is the report that at least one molybdoenzyme, namely arsenite oxidase, has crossed-over reduction potentials (Hoke et al., 2004): the Mo(V)/Mo(VI) couple has a more negative reduction potential than the Mo(IV)/Mo(V) couple, which indicates that electron bifurcation might not be restricted to flavins and the benzo- and menaquinones (Nitschke and Russell, 2012). It should be considered, however, that a short lived semiquinone or Mo(V) alone is not sufficient to allow productive electron bifurcation (Hoben et al., 2017).

It has frequently been stated that mechanisms cannot be proven. But we can certainly rule out many reasonable alternatives (Buskirk and Baradaran, 2009). One such alternative mechanism with non-crossed over reduction potentials was put forth for NfnAB before structural- and EPR spectroscopic studies had been performed (Wang et al., 2010). If the electron-bifurcating flavoprotein would have non-crossed reduction potentials, as in the case of flavodoxins (Alagaratnam et al., 2005), then the electron to leave the fully reduced flavoprotein first would have to be the one with a more negative reduction potential and the electron to leave second would have to be the one with the more positive reduction potential. In this case a stable flavin radical as intermediate should have been observed, which was not so for NfnAB.

## Flavin-based electron bifurcation and molecular oxygen

The enzyme systems catalyzing flavin-based electron bifurcation differ in their oxygen sensitivity. The butyryl-CoA dehydrogenase-EtfAB complex, which contains no iron-sulfur- clusters, can be purified under oxic conditions and can even be tested in the presence of molecular O_2_, which can substitute for ferredoxin in picking up the low reduction potential electron generated by bifurcation. The product formed is the superoxide anion radical ${\text{O}}_{2}^{-}$, which disproportionates to H_2_O_2_ and O_2_ (Chowdhury et al., 2015). All the other electron-bifurcating enzyme complexes contain iron-sulfur-clusters (Table 1) and have to be purified and tested under strictly anoxic conditions. The experience is that the more iron-sulfur- clusters the complexes have, the more sensitive they are toward O_2_. For example, the MvhADG-HdrABC complex with 13 [4Fe-4S]-clusters and one [2Fe-2S]-cluster (Wagner et al., 2017), is the most O_2_ sensitive; the enzyme complex is almost instantly inactivated by only trace amounts of O_2_ (Kaster et al., 2011). By contrast, the FixABCX complex, which contains only two [4Fe-4S]-clusters, is active in nitrogen-fixing *A. vinelandii* growing under microaerophilic conditions (Ledbetter et al., 2017).

## Energy conservation associated with flavin-based electron bifurcation

**Figure 4:**
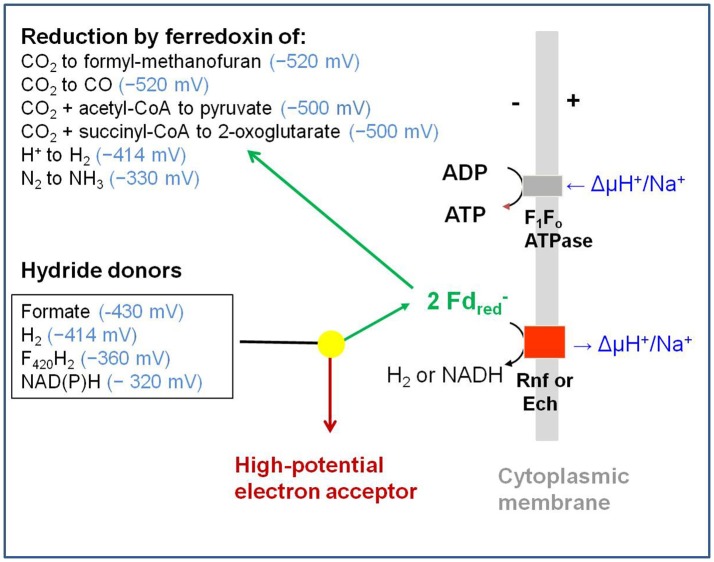
Electron-bifurcating reactions catalyzed by flavoenzyme complexes (yellow dot) and ferredoxin re-oxidizing reactions. The reduction potential E_0_′ of ferredoxin (Fd) is generally given as −420 mV, but it can be significantly lower (see Introduction). The E_0_′ of the other electron donors or acceptors are given in parenthesis. Note that the reduction potential E′ of the electron donors or acceptors under *in vivo* conditions (E′) may be quite different from E_0_′. Thus, E′ of ferredoxin can be lower than −500 mV, that of H_2_ as high as −300 mV and that of NADH is higher than that of NADPH. The electron-bifurcating flavoenzymes are cytoplasmic except for the enzyme complex that uses ubiquinone as a high potential electron acceptor. In the case of protons, or NAD(P)^+^ or pyruvate as high-potential electron acceptors, the electron-bifurcating reactions proceed reversibly: the back reaction is confurcating.

The term energy conservation is used in biochemistry when chemical energy or light energy is conserved in the form of ATP. The mechanisms involved are substrate-level phosphorylation, electron-transport phosphorylation or photophosphorylation with “energy-rich” intermediates that are in equilibrium with the ATP system. “Energy rich” in this sense are e.g., 1, 3-bisphosphoglycerate, phosphoenol pyruvate and acetyl phosphate in the case of substrate-level phosphorylation and the electrochemical proton- or sodium ion potential in the case of electron-transport phosphorylation. In aerobes with a respiratory chain, also NADH can be considered as “energy rich” and in anaerobes with an Rnf- and/or an Ech-complex also reduced ferredoxin or flavodoxin are “energy-rich,” because these electron donors are in equilibrium with the ATP-generating system via ΔμH^+^/Na^+^. Therefore, *stricto sensu*, flavin-based electron bifurcation is not a novel mechanism of energy conservation as has previously been proposed, but is a novel mechanism to generate the “energy-rich” reduced ferredoxin or flavodoxin, which via NAD^+^ or protons as electron acceptors can drive the phosphorylation of ADP (Figure 4).

The “energy rich” reduced ferredoxin is not only used for energy conservation via the Rnf- and/or Ech complex but also in reduction reactions with an E_0_′ more negative than −400 mV (Figure 4). An exception is the nitrogenase reaction with a reduction potential E_0_′ of −330 mV. Nitrogen reduction to NH_3_ has such a high activation energy that besides reduced ferredoxin additionally ATP is required.

## Evolutionary considerations

The electron-bifurcating flavoprotein complexes known to date fall into four phylogenetically distinct groups: (i) complexes containing EtfAB or EtfAB homologs with each subunit harboring an FAD; (ii) NfnAB complex with each subunit harboring an FAD; (iii) complexes containing an FMN-harboring NAD(P)H dehydrogenase subunit, with sequence similarity to NuoA of complex I from the *E. coli* respiratory chain; and (iv) complexes containing HdrABC with HdrA harboring FAD (Table 1). Flavin-based electron bifurcation has therefore evolved at least four times independently, which reflects the intrinsic electron-bifurcating properties of flavins and indicates an early evolution of this coupling mechanism (Martin, 2012; Nitschke and Russell, 2012; Barge et al., 2014; Lane, 2015).

Although genes encoding homologs of EtfAB-, HdrABC- or NuoA in anaerobic microorganisms are indicative for flavin- based electron bifurcation, their presence is not sufficient for this conclusion. For example, the acrylyl-CoA reductase-EtfAB complex from *Clostridium propionicum* (Hetzel et al., 2003; Kandasamy et al., 2013) appears not to be electron bifurcating (Buckel and Thauer, in press) although the reduction potential of the acrylyl-CoA/propionyl-CoA couple is almost 80 mV (E_0_′ = +70 mV) more positive than that of the crotonyl-CoA/butyryl-CoA couple (E_0_′ = −10 mV) (Sato et al., 1999) and although both EtfA- and EtfB-subunits contain FAD as a prosthetic group, which is a characteristic of electron-bifurcating Etfs as outlined before. NfnA shows sequence similarity to ferredoxin: NADP reductases of plants and bacteria but apparently these reductases are not electron bifurcating. Whereas NfnAB1 from *P. furiosus* is electron bifurcating, NfnAB2 from *P. furiosus* appears not to be (Nguyen et al., 2017). Homologs of the NADH dehydrogenase subunit NuoF are also found in many enzyme complexes that are not electron bifurcating. This includes the [FeFe]-hydrogenase HydABC from *S. wolfei*, which catalyzes the reduction of protons with NADH in the absence of reduced ferredoxin (Losey et al., 2017). Outside the methanogenic archaea, some of the HdrABC-containing enzyme complexes are probably not electron bifurcating. Thus, in evolution, the transition of non-electron-bifurcating flavoproteins to electron-bifurcating flavoproteins and *vice versa* probably was a frequent event.

## Flavin-based electron bifurcation and autotrophic CO_2_ fixation

A presently much discussed hypothesis is that the first living organisms on earth were chemolithoautotrophs capable of synthesizing all or most of their carbon compounds from CO_2_ using H_2_ or another inorganic electron donor as reductants (Sousa et al., 2013). The chemolithoautotrophic origin of life was spearheaded by Günter Wächtershäuser and is supported by experimental observations that in the presence of sufficient reducing power (E_0_′ = < −500 mV) individual CO_2_ fixation reactions involved in autotrophic CO_2_ fixation proceed spontaneously (Drobner et al., 1990; Wächtershäuser, 2016). Consistently, at least six different biological ways of autotrophic CO_2_ fixation exist that have independently evolved (Fuchs, 2011; Mall et al., 2018; Nunoura et al., 2018). They all involve ferredoxin-dependent reactions. In early biochemical evolution, flavin-based electron bifurcation might have been the simplest means to provide the required reducing power for ferredoxin reduction. The argument is that ferredoxin reduction via proton- or sodium ion motive force-driven reversed electron transport involving ATP hydrolysis and the Rnf- or Ech complexes (see Figure 4) requires a much higher organization level than flavin-based electron bifurcation (Goldford et al., 2017; Martin and Thauer, 2017). It has recently been proposed that metal iron Fe(0) could have been the electron donor for primordial CO_2_ reduction before flavin-based electron bifurcation came into play (Sousa et al., 2018).

## Electron bifurcation, a special form of sequential electron transport

Oxidation-reduction of most biological substrates involves pair wise exchange of electrons. Despite this fact electron transfer between redox centers occur one at a time when the two centers are further apart than in hydride transfer distance (3–5 Å) (Basner and Schwartz, 2004). This sequential transfer leads to the formation of free radicals. Already Leonor Michaelis (1875–1949) formulated that “the task of the protein may be to establish geometric configurations between different prosthetic groups and aid in the formation and stabilization of free radicals” (Michaelis, 1951). His view was that these radicals would form upon transfer of a single electron in a thermally activated endergonic step; a second, now exergonic electron transfer would then complete the overall two-electron oxidation or reduction of substrate (Page et al., 1999). In other words, the two electrons transferred are energetically not equivalent and reach their goal sequentially. In case of electron bifurcation the situation is similar but with the difference that the two electrons reach different goals. Therefore the foundations for electron bifurcation were laid already in the first half of the twentieth century.

## Outlook

Since 2008, the year of the discovery of flavin-based electron bifurcation, more than 10 enzyme systems that couple endergonic ferredoxin/flavodoxin-reducing reactions to exergonic redox reactions have been characterized (Table 1). In many anaerobes the reduced ferredoxin thus generated is used for energy conservation (Figure 4). Genome-sequence comparisons indicate that only the tip of the iceberg has been seen.

In 1975, Peter Dennis Mitchell proposed ubiquinol as the site of electron bifurcation in the cytochrome *bc*_1_ complex of mitochondria (Mitchell, 1975a,b, 1976). This proposal was subsequently supported by many experiments and revolutionized our thinking of how energy is conserved in chemotrophic and phototrophic aerobes. Flavin-based electron bifurcation differs from ubiquinol-/menaquinol-/plastoquinol-based electron bifurcation in that it is mostly associated with the cytoplasm and operates at reduction potentials almost 400 mV more negative. “It is probably safe to say that the importance of flavin-based electron bifurcation for understanding energy coupling in the anaerobic world is as important as ubiquinol-/menaquinol-/ plastoquinol-based electron bifurcation is for understanding energy coupling in the aerobic chemotrophic and phototrophic world” (Thauer, 2015).

## Author contributions

All authors listed have made a substantial, direct, and intellectual contribution to the work, and approved it for publication.

### Conflict of interest statement

The authors declare that the research was conducted in the absence of any commercial or financial relationships that could be construed as a potential conflict of interest.
